# Effectiveness of real-time polymerase chain reaction assay for the detection of *Mycobacterium tuberculosis* in pathological samples: a systematic review and meta-analysis

**DOI:** 10.1186/s13643-017-0608-2

**Published:** 2017-10-25

**Authors:** Emmanuel O. Babafemi, Benny P. Cherian, Lee Banting, Graham A. Mills, Kandala Ngianga

**Affiliations:** 1 Microbiology Department, Pathology Division, Sandwell and West Birmingham Hospitals NHS Trust, Birmingham, UK; 2 Microbiology Department, Bart’s Health NHS Trust, London, UK; 3 School of Pharmacy and Biomedical Sciences, University of Portsmouth, Portsmouth, UK; 4 School of Health Sciences and Social Work, University of Portsmouth, Portsmouth, UK

**Keywords:** Tuberculosis, *Mycobacterium tuberculosis*, Real-time polymerase chain reaction assay, Pulmonary samples, Extra-pulmonary samples, Systematic review, Meta-analysis

## Abstract

**Background:**

Rapid and accurate diagnosis of tuberculosis (TB) is key to manage the disease and to control and prevent its transmission. Many established diagnostic methods suffer from low sensitivity or delay of timely results and are inadequate for rapid detection of *Mycobacterium tuberculosis* (MTB) in pulmonary and extra-pulmonary clinical samples. This study examined whether a real-time polymerase chain reaction (RT-PCR) assay, with a turn-a-round time of 2 h, would prove effective for routine detection of MTB by clinical microbiology laboratories.

**Methods:**

A systematic literature search was performed for publications in any language on the detection of MTB in pathological samples by RT-PCR assay. The following sources were used MEDLINE via PubMed, EMBASE, BIOSIS Citation Index, Web of Science, SCOPUS, ISI Web of Knowledge and Cochrane Infectious Diseases Group Specialised Register, grey literature, World Health Organization and Centres for Disease Control and Prevention websites. Forty-six studies met set inclusion criteria. Generated pooled summary estimates (95% CIs) were calculated for overall accuracy and bivariate meta-regression model was used for meta-analysis.

**Results:**

Summary estimates for pulmonary TB (31 studies) were as follows: sensitivity 0.82 (95% CI 0.81–0.83), specificity 0.99 (95% CI 0.99–0.99), positive likelihood ratio 43.00 (28.23–64.81), negative likelihood ratio 0.16 (0.12–0.20), diagnostic odds ratio 324.26 (95% CI 189.08–556.09) and area under curve 0.99. Summary estimates for extra-pulmonary TB (25 studies) were as follows: sensitivity 0.70 (95% CI 0.67–0.72), specificity 0.99 (95% CI 0.99–0.99), positive likelihood ratio 29.82 (17.86–49.78), negative likelihood ratio 0.33 (0.26–0.42), diagnostic odds ratio 125.20 (95% CI 65.75–238.36) and area under curve 0.96.

**Conclusions:**

RT-PCR assay demonstrated a high degree of sensitivity for pulmonary TB and good sensitivity for extra-pulmonary TB. It indicated a high degree of specificity for ruling in TB infection from sampling regimes. This was acceptable, but may better as a rule out add-on diagnostic test. RT-PCR assays demonstrate both a high degree of sensitivity in pulmonary samples and rapidity of detection of TB which is an important factor in achieving effective global control and for patient management in terms of initiating early and appropriate anti-tubercular therapy.

**Systematic review registration:**

PROSPERO CRD42015027534.

**Electronic supplementary material:**

The online version of this article (10.1186/s13643-017-0608-2) contains supplementary material, which is available to authorized users.

## Background

Tuberculosis (TB), an infectious disease caused by the bacillus *Mycobacterium tuberculosis* (MTB), is spread from person to person predominantly through an airborne route. It remains a major global health problem as it causes ill-health among millions of people. After the human immunodeficiency virus (HIV), TB ranks as the second leading cause of death from an infectious disease worldwide [1, 2]. The lack of a simple and effective diagnostic test that can be utilised in resource-limited settings, where the infection is endemic, has hindered its control [3]. According to the World Health Organization in 2015, there were 10.4 million new cases of TB worldwide that resulted in 1.8 million deaths and over 95% were from low- and middle-income countries [4, 5]. In the UK, a total of 5758 TB cases were notified the by Public Health England in 2015 [6].

Over the past decade, the TB diagnostics pipeline has expanded, with several technologies showing promise [7]. New diagnostic tests are continually being developed, driven by demands for improvements in speed, cost, ease of use, patient safety and diagnostic accuracy [8]. Consequently, there are often several tests available for the diagnosis of a condition. Polymerase chain reaction (PCR) technology was introduced in the mid-1990s and has revolutionised the diagnosis of infectious diseases. Nucleic acid amplification assays (NAAAs) are commonly used in routine laboratories in industrialised countries for rapid and specific detection of MTB complex in clinical specimens. Over time, a significant improvement of PCR technologies has been achieved with the development of real-time polymerase chain reaction (RT-PCR) assay testing platforms. RT-PCR assay is commonly used to determine whether DNA or a sequence of the MTB is present in a sample and detects amplified DNA as the reaction progresses in real time. It monitors the amplification of a targeted DNA/RNA molecule during the PCR amplification by using complementary primers, i.e. in real-time, and not at its end, as in conventional PCR. A RT-PCR assay uses marked probes with fluorophores that emit fluorescence alongside amplification. The cycle of the PCR protocol in which there appears significant fluorescence is proportional to the quantity of DNA/RNA present in the sample. This value is called cycle threshold (*C*_t_) or cycle quantification (*C*_q_). RT-PCR is sensitive, specific and reproducible, and automation of the procedure reduces hands-on time and decreases the risk of cross-contamination [9].

RT-PCR-based methods have been shown to detect MTB with higher sensitivity and specificity directly from positive cultures or clinical specimens within 2 h [10]. It requires approximately 6 copies/ml of MTB DNA in comparison to smear microscopy that requires 5000–10,000 bacilli/ml. For concentrated samples, such as sputum, sensitivity of smear microscopy has been reported to increase up to 39%. Culture which is the reference standard, requires at least 100 viable bacilli to obtain a positive culture with a turn-a-round time of between 2 and 10 weeks [11]. Therefore, to adequately treat and effectively control MTB, there is a need for effective, rapid and accurate diagnosis.

This review assesses all the available published primary research studies to provide summary estimates of the effectiveness of RT-PCR assay for the detection of MTB from pulmonary and extra-pulmonary samples. It summarises current evidence-based clinical practice that can help to develop future guidelines and healthcare policy when choosing the most appropriate tool for rapid and accurate detection of MTB in pathological samples on routine basis.

## Methods

This review is in accord with the standardised written protocol (systematic review registration: PROSPERO CRD42015027534) that followed the PRISMA (Preferred Reporting Items for Systematic Reviews and Meta-Analyses) statement guidelines [12]. Additional file 1 shows the PRISMA checklist. Quality of included studies was assessed by Quality Assessment of Diagnostic Accuracy Studies-2 (QUADAS-2) [13]. Institutional ethical review approval was not needed for this review.

### Strategy

#### Electronic searches

Search terms (included ‘tuberculosis’, ‘*Mycobacterium tuberculosis*’, ‘pulmonary tuberculosis’, ‘extra-pulmonary tuberculosis’, ‘real-time polymerase chain reaction’, ‘RT-PCR’, ‘nucleic acid amplification test’, ‘culture-based media’, ‘liquid media’ and ‘solid media’) (see Additional file 2 for search terms) were used to generate a list of primary studies in any language from January 1995 to November 2016 (RT-PCR became a tool for detecting and quantifying expression profiles of selected genes in the mid-1990s. A search using the key words real-time and PCR yielded seven publications in 1995). Two investigators (EB, BC) independently and systematically carried out the search. Searches using electronic bibliographic databases (MEDLINE via PubMed, EMBASE, BIOSIS Citation Index, Web of Science, SCOPUS, ISI Web of Knowledge, Cochrane Infectious Diseases Group Specialised Register (CIDG SR), Cochrane Registry of Diagnostic Studies, National Institute for Health Research, PROSPERO, Google Scholar Turning Research into Practice (TRIP) and International Union Against Tuberculosis and Lung Disease (IUALTD)) took place in July 2015 and was updated in November 2016. The MEDLINE search strategy is outlined in Additional file 2. The MEDLINE search was imported to EMBASE, Cochrane Infectious Diseases Group Specialised Register and other databases to identify additional records [14, 15]. These attempts to avoid missing studies achieve a more reliable estimate of diagnostic accuracy which is important to ensure that the process of identifying studies is as thorough and unbiased as possible.

We reviewed reference lists of included articles and any relevant review articles identified through the above methods. Conference Proceedings Citation Index-Science (CPCI-S) was searched. We searched the portal of the World Health Organization (WHO) International Clinical Trials Registry Platform (http://www.who.int/trialsearch), to identify ongoing trials and StopTB Partnership’s New Diagnostics Working Group (www.stoptb.org/wg/new_diagnostics/). Personal communication was sent to the corresponding author of ‘Detection of Mycobacterial DNA directly from FNAC samples of tuberculous lymphadenopathy using real-time PCR: a preliminary study’ to ask for study data. Forward citation searching of relevant articles using the PubMed related articles and relevant guidelines (i.e. National Institute for Health and Care Excellence (NICE) in the UK) was performed. Focus was placed on TB meetings for example annual scientific conferences of TB diagnosis and control such as the IUALTD. Besides full articles, abstracts and letters to the editor with sample sizes >20 were also considered for inclusion. There was no language limitation to the search. Abstracts or articles in languages other than English were screened using ‘Google Translator’.

### Inclusion and exclusion criteria

Study designs such as cross-sectional studies, cohort studies (prospective and retrospective) and case-control designs for the detection of MTB from human pathological samples of any patient age were eligible for inclusion if the studies (1) described original research, (2) compared RT-PCR assay to a reference/gold standard method— culture-based (either liquid or solid) assay, (3) reported total number of patients tested and positive/negative results that allowed calculation of true positives (TP), true negatives (TN), false positives (FP) and false negatives (FN) and (4) were published between 1995 and 2016 in any language. Studies were excluded if (1) all samples were not tested by reference/gold standard test—culture-based (either liquid or solid) assay, (2) application of RT-PCR assay for determining drug resistance, (3) RT-PCR assay was not used in the study, (4) reference test was a combination of greater than one diagnostic test, (5) it included animal studies, (6) RT-PCR assay was used for detecting non-tuberculosis mycobacteria, (7) RT-PCR assay was used for detecting MTB from clinical isolates and not the pathological specimens/samples and (8) possible duplicate publication, when an author published more than one study. The existence of overlapping study populations was ascertained by checking sample recruitment sites and/or periods. The article reporting on the largest number of samples was included in our study.

### Selection of studies

Full-text articles were screened independently (by EB and BC, using a PRISMA flow chart [12]) for eligibility for use in the study to minimise bias in selection. Any disagreements were resolved through discussion and where needed, by a third reviewer. Any rejected studies were documented.

### Data extraction

Data were extracted (independently by EB and BC) from each selected study using a predetermined list of categories/characteristics: participants/population, index test, reference test, country, disease and target sequence for detection of MTB DNA (Table 1).

**Table 1:** Characteristics of the included studies

Author year [n]	Country	Total number of samples (*N*)		Reference test: culture	Index test RT-PCR	Target sequence
		PTB	EPTB			
Albuquerque, 2014 [24]	Brazil	140	–	LJ 7H9	TaqMan	IS6110 gene
Antonenka, 2013 [25]	Germany	116	–	MGIT LJ	TaqMan	rpoB as target sequence
Armand, 2011 [26]	France	70	47	LJ BacT/Alert MP	TaqMan	IS6110 gene
Barletta, 2014 [27]	Belgium	112	–	LJ	Light Cycler 480 Real-time PCR assay	IS6110 gene
Bloemberg, 2013 [28]	Switzerland	829	280	7H11 MGIT	COBAS TaqMan	16S rRNA gene
Causse, 2011 [23]	Spain	–	340	LJ 7H9	COBAS TaqMan MTB	16S rRNA gene
Chadran, 2010 [29]	India	72	–	LJ MGIT 960	COBAS TaqMan	IS6110 gene
Chaidir, 2012 [30]	Indonesia	–	207	Ogawa egg medium MB/BacTalert	IS6110-PCR BioRad	IS6110 gene
Chang, 2015 [31]	South Korea	2859	–	MGIT 960 3% Ogawa	AdvanSure TB/NTM Real-time PCR assay	IS6110 gene
Chen, 2012 [32]	China	178	–	BACTEC™ MGIT™ 960	Real-time PCR assay used the ABI Prism SDS 7000	IS6110 gene
Chitnis, 2010 [33]	India	–	204	LJ MGIT-BACTEC	Geno-Sen’s MTB complex Real-time PCR assay	16S rRNA gene
Cho, 2015 [34]	South Korea	2384	626	2% Ogawa medium MGIT 960	COBAS TaqMan MTB assay	IS6110 gene
Choe, 2011 [35]	South Korea	–	129	3% Ogawa	TaqMan	Real-time MTB PCR, targeting the senX3-regX3 intergenic region
Choi, 2013 [36]	South Korea	360	65	MGIT 960	COBAS TaqMan MTB assay	IS6110 gene
Dayal, 2010 [37]	India	–	47	BacT/Alert	Real-time PCR targeting 16SrRNA using Light Cycler RNA amplification syber green 1 kit (Roche Applied Biosciences, Germany)	16S rRNA gene
El Khechine, 2009 [38]	France	–	134	BACTEC 9000 MB LJ	Not specified	IS6110 gene
Feizabadi, 2012 [39]	Iran	247	–	LJ	TaqMan	Cytochrome P450 Cyp 141 gene
Friedrich, 2011 [40]	South Africa	–	25	MGIT 960	Xpert	*rpo*B probe
Gous, 2012 [41]	South Africa	–	39	BACTEC 9000 MB LJ	Light Cycler mycobacterium detection assay (LCTB)	
Hillemann, 2011 [42]	Germany	–	521	MGIT 960 LJ	GenoType MTBC and CM/AS assays (Hain Lifescience)	*rpo*B probe
Hofmann-Thiel, 2016 [43]	Germany	608	107	MGIT LJ	Abbott Real-time MTB	Antigen b (*PAB*) and the multicopy insertion element IS6110 gene
Huh, 2015 [44]	South Korea	6852	–	MGIT 960 LJ	COBAS TaqMan	16S rRNA gene
In, 2014 [45]	South Korea	247	–	BACTEC MGIT 960 LJ	Ultrafast NBS LabChip G2–3 (NanoBioSys)	
Jönsson, 2015 [46]	Sweden	2388	1005	MGIT 960 LJ	COBAS TaqMan	16S rRNA gene
Kheawon, 2012 [47]	Thailand	430	–	LJ medium	Commercial PCR Kits Amplicor	IS6110 gene and MPB64 gene
Kim, 2011 [48]	South Korea	96	310	Solid culture	Ultrafast NBS LabChip G2–3 system (NanoBioSys)	61 genomic DNA (gDNA) samples of MTB
Lee, 2011 [49]	South Korea	99	–	3% Ogawa	AdvanSure TB/NTM Real-time PCR assay	IS6110 gene
Lee, 2010 [50]	South Korea	–	143	3% Ogawa	Light Cycler 2.0	senX3-regX3 intergenic region
Lee, 2013 [51]	Taiwan	587	–	BACTEC MGIT 960 LJ	COBAS TaqMan MTB assay	IS6110 gene
Lim, 2014 [52]	South Korea	1167			COBAS TaqMan MTB assay	
Linasmita, 2012 [53]	Thailand	–	73	MGIT 960	COBAS TaqMan MTB assay	16S ribosomal RNA gene of *M. tuberculosis*
Lira, 2013 [54]	Brazil	165	–	LJ	ABI Prism 7500 Sequence Detection System (Applied Biosystems) using TaqMan-specific probe	IS6110 gene
Luo, 2010 [55]	USA	–	70	Culture-based assay (type not stated)	SmartCycler II instrument	IS6110 gene
Malhotra, 2012 [56]	India	–	555	7H9	COBAS Taqman	IS6110 gene
Mangat, 2016 [57]	India	74	–	MGIT 960 LJ	Roche Light Cycler 480 Real-time PCR system	123 bp fragment of insertion element IS6110 sequence
Miller, 2011 [58]	North Carolina, USA	89	23	7H9 LJ	Xpert	Laboratory-developed test targeting IS6110 gene
Moure, 2012 [59]	Spain	–	149	MGIT 960 LJ	GX assay	
Park, 2013 [60]	South Korea	320	–	MGIT 960 3% Ogawa	COBAS TaqMan	IS6110 gene
Pinhata, 2015 [61]	Brazil	715	–	MGIT 960 Ogawa–Kudoh slant	Roche Light Cycler 480 II system	mpt64 gene
Rachow, 2011 [11]	Tanzania	292	–	Both liquid and solid (type not stated)	Cepheid Xpert MTB/RIF assay	rpoB as target gene
Rosso, 2011 [62]	Brazil	–	158	LJ	ABI Prism 7500 system (Applied Biosystems)	IS6110 gene
Sethi, 2012 [63]	India	50	22	MGIT 960 LJ	In-house mpt64 Real-time PCR	mpt64 gene
Sharma, 2015 [64]	India	1480	–	MGIT 960 LJ	ABI prism 3130xl genetic analyser (Applied Biosystems)	81-bp rpoB gene
Tortoli, 2012 [65]	Italy	4340	1727	MGIT 960 LJ	Cepheid Xpert MTB/RIF assay	rpoB as target gene
Wang, 2013 [66]	China	30	–	Bact/Alert 3D	Light Cycler 480 (Roche)	
Yang, 2011 [67]	Taiwan	1093	–	MGIT 960 LJ 7H11	COBAS TaqMan MTB assay	IS6110 gene

Key: *LJ* Löwenstein-Jensen, *Middlebrook 7H9 Broth* Liquid growth medium, *Middlebrook 7H11* Solid medium, *MGIT* Mycobacterium Growth Indicator Tube, *PTB* Pulmonary TB, *EPTB* Extra-pulmonary TB, *[n]* reference list number

### Assessment of study quality

The methodological quality for the included studies was assessed independently (EB and BC) according to the four domains (patient selection, index test, reference standard and flow and timing) of the QUADAS-2 tool [13]. The study QUADAS-2 quality criteria are given in Additional file 3.

### Data synthesis and meta-analysis

For each study, we computed measures of test accuracy using standard methods recommended for meta-analysis of diagnostic studies: sensitivity, specificity, positive likelihood ratio (PLR), negative likelihood ratio (NLR), diagnostic odds ratio (DOR) and 95% confidence intervals (CI) [16–18] see Additional file 4. TP, FP, TN and FN were extracted directly from source papers. Where this information was not available, values were calculated from the data provided in the article. To assess the overall accuracy, a DOR was calculated using the DerSimonian-Laird random-effect model, which accounts for both within-study variability (random error) and between-study variability (heterogeneity) along with the area under the summary receiver operating characteristic (SROC) curve using the bivariate model [17, 18]. The bivariate model considers potential threshold effects and the correlation between binary tests (sensitivity and specificity). These measures were pooled using the random-effects model [17, 18]. Each study in the meta-analysis contributed a pair of numbers: sensitivity and specificity. Since these measures are correlated, we summarised their joint distribution using a SROC curve. The SROC curve presents a global summary of test performance and shows the trade-off between sensitivity and specificity. A symmetric curve suggests that the variability in accuracy between studies is explained, in part, by differences in thresholds used by the studies. The area under the SROC curve is a global measure of overall performance of the test. An area under the curve value of 1 indicates perfect discriminatory ability of the test, while an area under the curve value of 0.5 means that the test does not have discriminating ability [17, 18].

Data were analysed using Meta-DiSC (version 1.4), Reviewing Manager ver. 5.3 (Cochrane Collaboration, Oxford, UK) [18, 19]. The data were displayed graphically on forest plots and SROC plots. The SROC curve was fitted using the Littenberg-Moses method [20].

Publication bias was not evaluated as this is not usually recommended in the meta-analysis for diagnostic test accuracy [21]. Generally, a diagnostic accuracy study does not test a hypothesis; therefore, there is no *p* value for authors and publishers that may influence decisions about publication which are based on the statistical significance of the results [22].

### Investigations of heterogeneity

Exploring heterogeneity is a critical issue (1) to understand the possible factors that influence accuracy estimates and (2) to evaluate the appropriateness of statistical pooling of accuracy estimates using random-effects meta-analysis to generate sensitivity and specificity with 95% CIs from various studies [22].

The heterogeneities among studies were assessed visually with forest plots and SROC curves with 95% prediction regions and statistically with chi-squared (*χ* ^2^) and using I-squared (*I* ^2^) statistics with the following interpretation: *I* ^2^ = 0, no heterogeneity; 0 < *I* ^2^ < 25, mild heterogeneity; 25 ≤ *I* ^2^ < 50, moderate heterogeneity; 50 ≤ *I* ^2^ < 75, strong heterogeneity; 75 ≤ *I* ^2^ < 90, considerable heterogeneity and 90 ≤ *I* ^2^, extreme heterogeneity [23]. Source of heterogeneity was investigated using stratified (subgroup) analyses. The following factors were specified a priori as potential sources of heterogeneity (1) studies of RT-PCR assay type: CobasTaqMan as the RT-PCR assay, Light Cycler as the RT-PCR assay, Cepheid & others and (2) RT-PCR assay target sequence gene: IS6110 as the RT-PCR assay target sequence gene, 16SRNA as the RT-PCR assay target sequence gene and other genes as the RT-PCR assay (see Tables 1, 3 and 4).

## Results

### Study characteristics

**Fig. 1:**
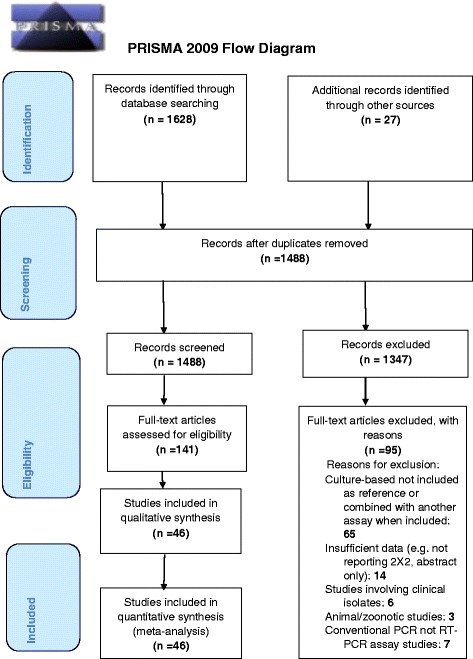
The Preferred Reporting Items for Systematic Reviews and Meta-Analyses (PRISMA)

Of the 6706 references that were identified initially, 1628 potentially relevant citations were selected based on relevance to the study topic. An additional 27 studies were identified from grey literature and references of full-text articles. After screening all the titles and abstracts, removing the duplicates and excluding the ineligible studies, 46 articles [10, 23–67] were selected for full-text review and meta-analysis (Fig. 1). Twenty-one [10, 24, 25, 27, 29, 31, 32, 39, 44, 45, 47, 49, 51, 52, 54, 57, 60, 61, 64, 66, 67] reported detection of pulmonary TB (PTB), fifteen [23, 30, 33, 35, 37, 38, 40–42, 50, 53, 55, 56, 59, 62] reported detection of extra-pulmonary TB (EPTB) and ten [26, 28, 34, 36, 43, 46, 48, 58, 63, 65] reported on both types of pathological sample. Table 1 summarises the main characteristics of the included studies. In total, the review and meta-analysis included 35,380 (28,406 PTB and 6974 EPTB) pathological samples obtained from 21 countries with high, moderate and low prevalence of TB. Studies included patients with infections identified in primary, secondary and tertiary healthcare settings. Details of the RT-PCR assays used are summarised in Table 1.

**Fig. 2:**
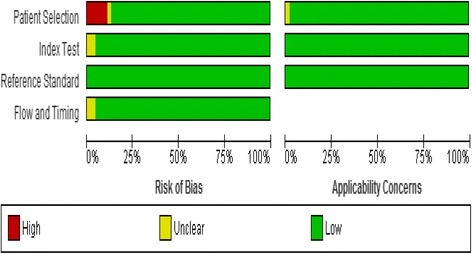
Risk of bias and applicability concerns graph: review authors’ judgements about each domain presented as percentages across included studies

**Fig. 3:**
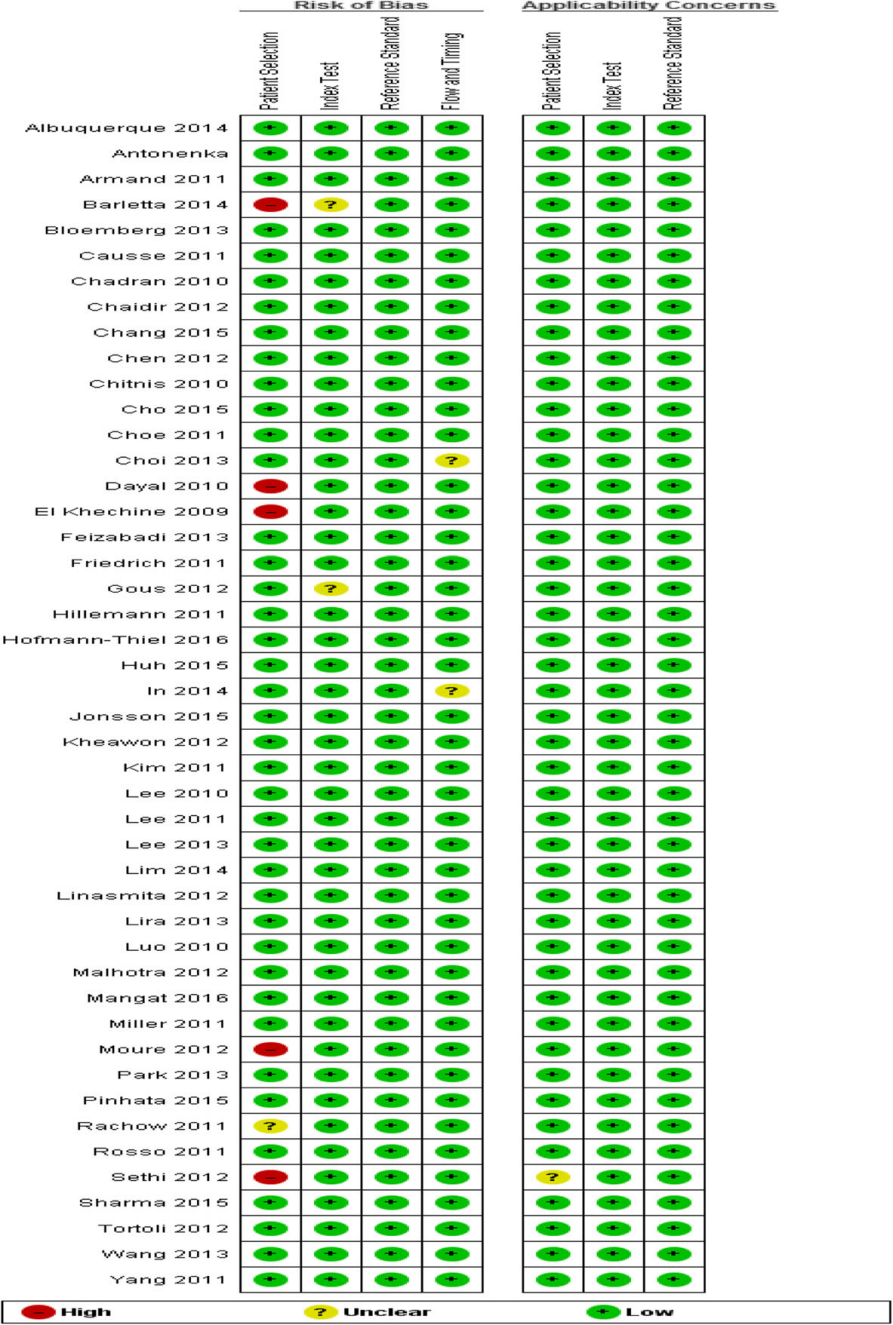
Risk of bias and applicability concerns summary: review authors’ judgements about each domain for each included study

The methodological quality of studies (assessed by the QUADAS-2 tool) was generally high, with 37 of the studies meeting all four domains of the criteria (see Figs. 2 and 3). All studies used RT-PCR assay as index test and culture-based assay as the reference test.

### Meta-analysis

**Fig. 4:**
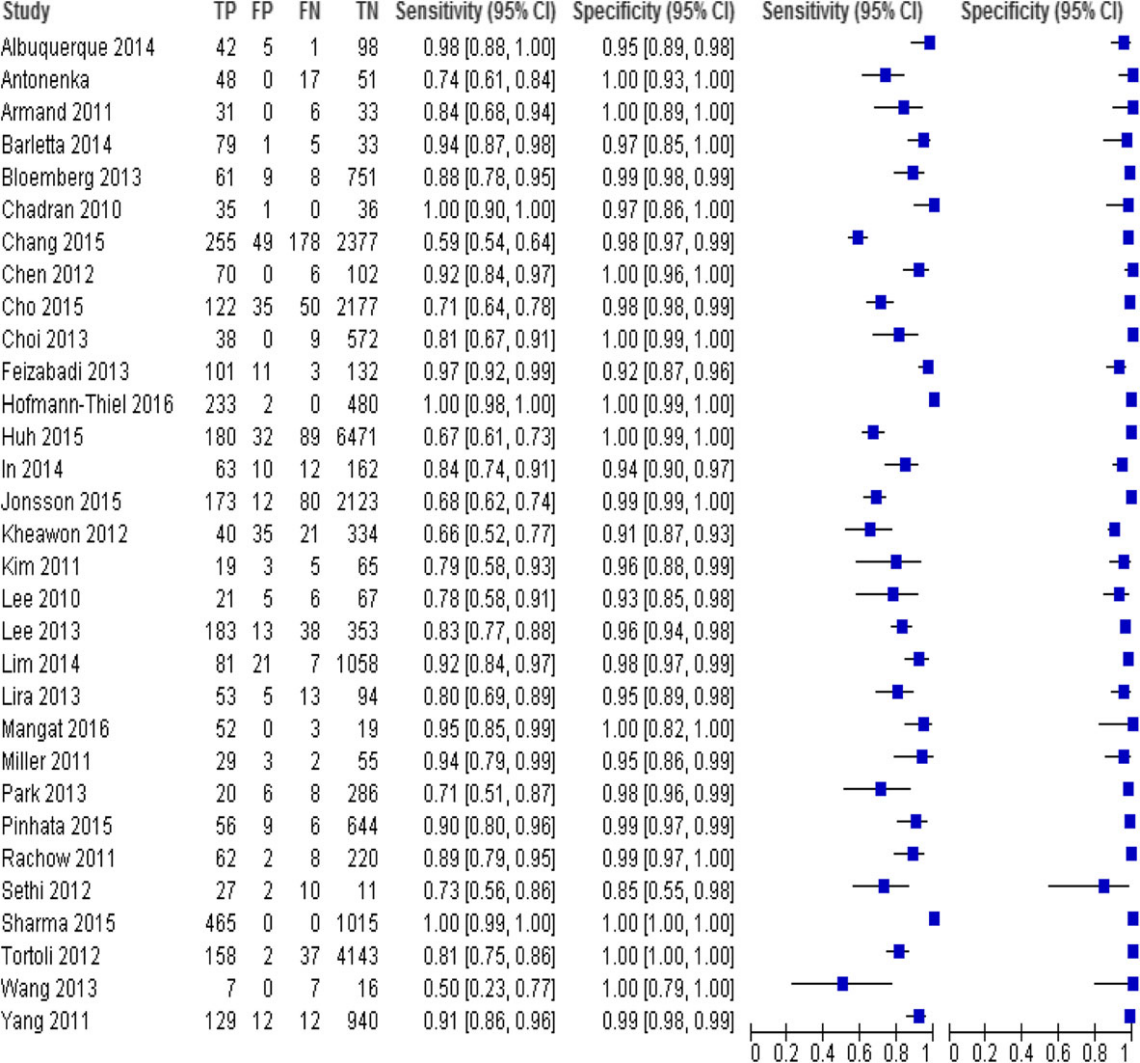
Forest plot of estimates of RT-PCR assay for pulmonary tuberculosis (PTB). TP = true positive, FP = false positive, FN = false negative, TN = true negative. Between brackets are the 95% CI of sensitivity and specificity. The figure shows the estimated sensitivity and specificity of the study (blue squares) and its 95% CI (black horizontal line)

**Fig. 5:**
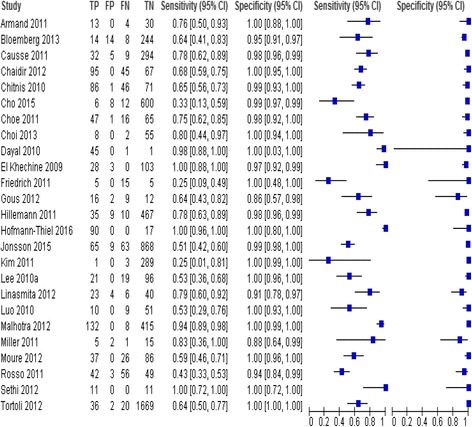
Forest plot of estimates of RT-PCR assay for extra-pulmonary tuberculosis (EPTB). TP = true positive, FP = false positive, FN = false negative, TN = true negative. Between brackets are the 95% CI of sensitivity and specificity. The figure shows the estimated sensitivity and specificity of the study (blue squares) and its 95% CI (black horizontal line)

Results as 95% CI values were as follows: overall sensitivity 0.82 (95% CI 0.81–0.83) and 0.70 (95% CI 0.67–0.72) and the values and confidence intervals for specificity are similar 0.99 (95% CI 0.99–0.99) for PTB and EPTB samples, respectively. AUC was 0.99 and 0.96 for PTB and EPTB samples, respectively. The summary estimates of PTB for heterogeneity with chi-squared (*χ* ^2^) using 95% CI for sensitivity, specificity, PLR, NLR and DOR were 586.21, 361.23, 285.01, 359.13 and 242.84, respectively, and *p* = 0 indicating significant heterogeneity across studies. *I* ^2^ was between 87.60 and 92.80% showing significant heterogeneity. The summary estimates of EPTB heterogeneity with chi-squared (*χ* ^2^) using 95% CI for sensitivity, specificity, PLR, NLR and DOR were 272.48, 105.48, 75.37, 186.30 and 70.73, respectively, and *p* = 0 indicating significant heterogeneity across studies. *I* ^2^ was between 66.10 and 91.20% showing significant heterogeneity. There were considerable heterogeneities (see Table 2, Figs. 4 and 5) in these data.

**Table 2:** Summary of statistical results for pulmonary tuberculosis (PTB) and extra-pulmonary tuberculosis (EPTB) pathological samples

Test property	Summary measure of test accuracy^a^ (95% CI)	Test for heterogeneity		
		(*χ* ^2^) (df = 24)	(*I* ^2^)	*p* value
PTB (*n* = 31; ^b^28,406) AUC = 0.99				
Sensitivity	0.82 (0.81–0.83)	586.21	92.8%	< 0.001
Specificity	0.99 (0.99–0.99)	361.23	91.7%	< 0.001
Positive likelihood ratio (PLR)	42.77 (28.23–64.81)	285.01	89.5%	< 0.001
Negative likelihood ratio (NLR)	0.16 (0.12–0.20)	359.13	91.6%	< 0.001
Diagnostic odds ratio (DOR)	324.26 (189.08–556.09)	242.84	87.6%	< 0.001
EPTB (*n* = 25; ^b^6974) AUC = 0.96				
Sensitivity	0.70 (0.67–0.72)	272.48	91.20%	< 0.001
Specificity	0.99 (0.99–0.99)	105.48	77.20%	< 0.001
Positive likelihood ratio (PLR)	29.82 (17.86–49.78)	75.37	68.20%	< 0.001
Negative likelihood ratio (NLR)	0.33 (0.26–0.42)	186.30	87.10%	< 0.001
Diagnostic odds ratio (DOR)	125.20 (65.76–238.36)	70.73	66.10%	< 0.001

*χ* ^2^ chi-squared, *df* degree of freedom, *I* ^2^ I-squared, *n* number of studies, *CI* confidence interval, *AUC* area under receiver operating characteristics curve, *PTB* pulmonary tuberculosis, *EPTB* extra-pulmonary tuberculosis

^a^Random-effects model

^b^Number of specimens

### Subgroup analyses by RT-PCR assay type

I. With CobasTaqMan (Table 1) as the RT-PCR assay (17 studies, 19,814 specimens), the results were as follows: sensitivity 0.78 (95% CI 0.76–0.80), specificity 10.99 (95% CI, 0.99–0.99) and AUC 0.98. A test with perfect discrimination has a ROC curve that passes through the upper left corner (100% sensitivity, 100% specificity). The closer the ROC curve to the upper left corner, the higher the overall accuracy of the test. The summary estimates of CobasTaqMan heterogeneity with chi-squared (*χ* ^2^) using 95% CI for sensitivity, specificity, PLR, NLR and DOR were 205.13, 127.21, 98.14, 134.36 and 47.6, respectively, and *p =* < 0.001 indicating significant heterogeneity across studies. *I* ^2^ was between 70.50 and 91.20% showing significant heterogeneity. The results for subgroup analysis by RT-PCR assay type are as presented in Table 3 and Additional file 5 and show considerable heterogeneity.
II. With Light Cycler (Table 1) as the RT-PCR assay (7 studies, 1159 specimens), the results were as follows: sensitivity 0.85 (95% CI 0.80–0.88), specificity 0.99 (95% CI 0.98–0.99) and AUC 0.97. A test with perfect discrimination has a ROC curve that passes through the upper left corner (100% sensitivity, 100% specificity). The closer the ROC curve to the upper left corner, the higher the overall accuracy of the test. The summary estimates of Light Cycler heterogeneity with chi-squared (*χ* ^2^) using 95% CI for sensitivity, specificity, PLR, NLR and DOR were 61.71, 10.09, 17.45, 63.77, 20.35 respectively and *p =* < 0.121 indicating significant heterogeneity across studies. *I* ^2^ was between 66.40 and 90.30% showing significant heterogeneity. The results for subgroup analysis by RT-PCR assay type are as presented Table 3 and Additional file 5 and show considerable heterogeneity.
III. With Cepheid and others (Table 1) as the RT-PCR assay (22 studies, 14,839 specimens), the results were as follows: sensitivity 0.78 (95% CI 0.77–0.80), specificity 0.99 (95% CI 0.99–0.99) and AUC 0.99. A test with perfect discrimination has a ROC curve that passes through the upper left corner (100% sensitivity, 100% specificity). The closer the ROC curve to the upper left corner, the higher the overall accuracy of the test. The summary estimates of Cepheid and others heterogeneity with chi-squared (*χ* ^2^) using 95% CI for sensitivity, specificity, PLR, NLR and DOR were 729.43, 299.13, 234.62, 594.30 and 208.25, respectively, and *p* = 0 indicating significant heterogeneity across studies. *I* ^2^ was between 89.90 and 97.10% showing significant heterogeneity. The results for subgroup analysis by RT-PCR assay type are as presented Table 3 and Additional file 5 and show considerable heterogeneity.

**Table 3:** Subgroup analyses by RT-PCR assay type

RT-PCR assay type	Summary measure of test accuracy^a^ (95% CI)	Test for heterogeneity		
		(*χ* ^2^) (df = 24)	(*I* ^2^)	*p* value
CobasTaqMan (*n* = 17; ^b^19,814) AUC = 0.98				
Sensitivity	0.78 (0.76–0.80)	205.13	92.20%	< 0.001
Specificity	0.99 (0.99–0.99)	127.21	87.40%	< 0.001
Positive likelihood ratio (PLR)	41.59 (27.80–62.18)	98.14	83.70%	< 0.001
Negative likelihood ratio (NLR)	0.18 (0.13–0.23)	134.36	88.10%	< 0.001
Diagnostic odds ratio (DOR)	273.14 (181.45–411.17)	47.6	66.40%	< 0.001
Light Cycler (*n* = 7; ^b^1159) AUC = 0.97				
Sensitivity	0.85 (0.80–0.88)	61.71	90.30%	< 0.001
Specificity	0.99 (0.98–0.99)	10.09	40.50%	0.121
Positive likelihood ratio (PLR)	21.65 (6.82–68.72)	17.45	65.60%	0.008
Negative likelihood ratio (NLR)	0.17 (0.08–0.38)	63.77	90.60%	< 0.001
Diagnostic odds ratio (DOR)	150.52 (31.97–708.78)	20.35	70.50%	< 0.002
Cepheid and others (*n* = 22; ^b^14,839) AUC = 0.99				
Sensitivity	0.78 (0.77–0.80)	729.43	97.10%	< 0.001
Specificity	0.99 (0.99–0.99)	299.13	93.00%	< 0.001
Positive likelihood ratio (PLR)	38.50 (19.65–75.42)	234.62	91.00%	< 0.001
Negative likelihood ratio (NLR)	0.22 (0.15–0.33)	594.30	96.50%	< 0.001
Diagnostic odds ratio (DOR)	221.44 (94.94–516.51)	208.25	89.90%	< 0.001

*χ* ^2^ chi-squared, *df* degree of freedom, *I* ^2^ I-squared, *n* number of studies, *CI* confidence interval, *AUC* area under receiver operating characteristics curve

^a^Random-effects model

^b^Number of specimens

### Subgroup analyses by RT-PCR assay target sequence gene

I. With IS6110 as the RT-PCR assay target sequence gene (22 studies, 12,004 specimens), the results were as follows: sensitivity 0.79 (0.77–0.81), specificity 0.98 (0.98–0.98) and AUC 0.99. A test with perfect discrimination has a ROC curve that passes through the upper left corner (100% sensitivity, 100% specificity). The closer the ROC curve to the upper left corner, the higher the overall accuracy of the test. The summary estimates of IS6110 heterogeneity with chi-squared (*χ* ^2^) using 95% CI for sensitivity, specificity, PLR, NLR and DOR were 470.30, 150.16, 134.64, 356.22 and 141.04, respectively, and *p* = 0 indicating significant heterogeneity across studies. *I* ^2^ was between 85.10 and 95.50% showing significant heterogeneity. The results for subgroup analysis by RT-PCR assay target sequence gene are presented in Table 4 and Additional file 5 and show considerable heterogeneity.
II. With 16S rRNA as the RT-PCR assay target sequence gene (7 studies, 12,074 specimens), the results were as follows: sensitivity 0.69 (0.66–0.71), specificity 0.99 (0.99–0.99) and AUC 0.97. A test with perfect discrimination has a ROC curve that passes through the upper left corner (100% sensitivity, 100% specificity). The closer the ROC curve to the upper left corner, the higher the overall accuracy of the test (*χ* ^2^) using 95% CI for sensitivity, specificity, PLR, NLR and DOR were 45.85, 32.10, 34.60, 19.87 and 9.12, respectively, and *p* < 0.167 indicating significant heterogeneity across studies. *I* ^2^ was between 86.90 and 34.20% showing significant heterogeneity. The results for subgroup analysis by RT-PCR assay target sequence gene are presented in Table 4 and Additional file 5 and show considerable heterogeneity.
III. With other genes (see Table 1) as the RT-PCR assay (17 studies, 11,870 specimens) the results were as follows: sensitivity 0.82 (0.80–0.84), specificity 0.99 (0.99–0.99) and AUC 0.98. A test with perfect discrimination has a ROC curve that passes through the upper left corner (100% sensitivity, 100% specificity). The closer the ROC curve to the upper left corner, the higher the overall accuracy of the test. The summary estimates of other genes heterogeneity with chi-squared (*χ* ^2^) using 95% CI for sensitivity, specificity, PLR, NLR and DOR were 413.02, 173.35, 123.92, 498.03 and 125.08, respectively, and *p* < 0.001 indicating significant heterogeneity across studies. *I* ^2^ was between 87.20 and 96.10% showing significant heterogeneity. The results for subgroup analysis by RT-PCR assay target sequence gene are presented in Table 4 and Additional file 5 and show considerable heterogeneity.

**Table 4:** Subgroup analyses by RT-PCR assay target sequence gene

RT-PCR assay genes	Summary measure of test accuracy^a^ (95% CI)	Test for heterogeneity		
		(*χ* ^2^) (df = 24)	(*I* ^2^)	*p* value
IS6110 (*n* = 22; ^b^12,004) AUC = 0.99				
Sensitivity	0.79 (0.77–0.81)	470.30	95.50%	< 0.001
Specificity	0.98 (0.98–0.98)	150.16	86.00%	< 0.001
Positive likelihood ratio (PLR)	31.76 (20.12–50.13)	134.64	84.40%	< 0.001
Negative likelihood ratio (NLR)	0.17 (0.12–0.24)	356.22	94.10%	< 0.001
Diagnostic odds ratio (DOR)	243.69 (127.07–437.37)	141.04	85.10%	< 0.001
16S rRNA (*n* = 7; ^b^12,074) AUC = 0.97				
Sensitivity	0.69 (0.66–0.72)	45.85	86.90%	< 0.001
Specificity	0.99 (0.99–0.99)	32.10	81.30%	< 0.001
Positive likelihood ratio (PLR)	67.64 (36.40–125.70)	34.60	82.50%	< 0.001
Negative likelihood ratio (NLR)	0.29 (0.24–0.36)	19.87	69.8%	0.003
Diagnostic odds ratio (DOR)	287.19 (193.85–425.46)	9.12	34.20%	0.167
Other genes (*n* = 17; ^b^11,870) AUC = 0.98				
Sensitivity	0.82 (0.80–0.84)	413.02	96.10%	< 0.001
Specificity	0.99 (0.99–0.99)	173.35	90.80%	< 0.001
Positive likelihood ratio (PLR)	42.48 (20.66–87.36)	123.92	87.10%	< 0.001
Negative likelihood ratio (NLR)	0.22 (0.13–0.37)	498.03	96.80%	< 0.001
Diagnostic odds ratio (DOR)	234.56 (86.01–639.63)	125.08	87.20%	< 0.001

*χ* ^*2*^ chi-squared, *df* degree of freedom, *I* ^*2*^ I-squared, *n* number of studies, *CI* confidence interval, *AUC* area under receiver operating characteristics curve, *IS6110 Mycobacterium tuberculosis* complex-specific insertion sequence, *16S rRNA* 16S ribosomal RNA gene of MTB, *Other genes* rpoB as target sequence, mpt64 gene, 81-bp rpoB gene, senX3-regX3 intergenic region, 61 genomic DNA (gDNA) samples of MTB, cytochrome P450 Cyp 141 gene

^a^Random-effects model

^b^Number of specimen

## Discussion

Tuberculosis is a global health threat and early and accurate diagnosis is crucial for preventing morbidity and mortality. Various methods are employed for the diagnosis of TB such as smear microscopy, culture identification, histopathology, tuberculin skin test (TST), serological assays, interferon-gamma release assays (IGRAs) and nucleic acid amplification (NAA) tests [68, 69]. Smear microscopy is widely used in the diagnosis of TB but has drawbacks owing to low and variable sensitivity values (0–40%) and cannot readily differentiate between MTB and non-tuberculous mycobacteria (NTM) [70–72]. Culture identification for MTB also has variable sensitivities (0–80%) in different TB specimens [63, 73–75] with turn-a-round time of 2–10 weeks requiring the use of skilful technicians [76]. Diagnosis of TB from tissue samples is usually made by histopathological examination that depends on the presence of granulomatous inflammation and caseous necrosis [70, 77]. However, histology does not distinguish between EPTB and infections from other granulomatous diseases such as NTM, sarcoidosis, leprosy and systemic lupus erythematosus (except for the presence of acid-fast bacilli (AFB)) [78, 79]. RT-PCR is a novel and robust assay primarily used to quantify the nucleic acids in all TB specimens [63, 80–82]. The main advantages of RT-PCR are shortened turn-a-round time, quantification of bacterial load and automation of the procedure that reduces hands-on time and decreased risk of cross-contamination [63, 83]. This review provides evidence on the effectiveness of RT-PCR assay for the rapid and accurate detection of MTB from pathological samples. To our knowledge, this is the first systematic review and meta-analysis for ascertaining the effectiveness of RT-PCR assays for the detection of MTB from both pulmonary and extra-pulmonary pathological samples.

In this study, results indicated that RT-PCR assay produces consistent results with high specificity of 0.99 (95% CI 0.99–0.99), PLR of 43.0 (28.23–64.81) and NLR of 0.16 (0.12–0.20) for PTB, whereas specificity, PLR and NLR were 0.99 (95% CI, 0.99–0.99), 29.82 (17.86–49.78) and 0.33 (0.26–0.42), respectively, for EPTB. A PLR of 43 suggests that patients with a pulmonary MTB infection have a 43-fold higher chance of being RT-PCR test positive compared with patients without the infection. This ratio suggests a potential role for RT-PCR assay in confirming (ruling in) a MTB infection.

The summary estimates of sensitivity, however, were 0.82 (95% CI 0.81–0.83) and 0.70 (95% CI 0.67–0.72) for pulmonary and extra-pulmonary samples, respectively, higher in pulmonary than extra-pulmonary TB possibly due to paucity of tubercle bacilli in extra-pulmonary samples. Sensitivity estimates were more variable than specificity. According to the AUC and the DOR (see Table 2), diagnostic accuracy of RT-PCR assay was excellent for the pulmonary specimens over extra-pulmonary and these results are acceptable for clinical practice (see Table 2).

A RT-PCR assay for the detection of MTB has a high sensitivity and specificity. The PLR and NLR showed that RT-PCR may serve as a suitable method when confirming or excluding TB. It was anticipated that there would be some degree of heterogeneity of diagnostic measures across studies due to differences in sample size, RT-PCR assay type, reference test (either liquid or solid or both) and TB prevalence. High heterogeneity was found among studies (as defined by the *χ* ^2^ and *I* ^2^ statistics) for all measures. Subgroup analyses were therefore performed pre-specified to investigate potential sources of the observed between-study heterogeneity. It was assumed that the disparity was likely a result of the differences in the type of index test (RT-PCR assay) or target sequence gene of MTB used.

In the current study, a limited number of subgroup analyses were conducted by comparing CobasTaqMan, Light Cycler, Cepheid and others as RT-PCR assay types to reduce the degree of study heterogeneity. Heterogeneity assessed by *χ* ^2^ and *I* ^2^ statistics between these subgroups was generally not very strong (see Table 3). However, significant heterogeneity of diagnostic accuracy measures was expected and was, indeed, found among studies and the random-effects model partially accounted for the between-study heterogeneity.

Some degree of heterogeneity of diagnostic measures across studies was found due to differences in sample size, study design, target genes and clinical settings of the participants. Thus, it is possible that when evaluating RT-PCR assays using a more sensitive index test can lead to overestimation of the assay’s sensitivity. No significant differences in specificities of the different types of index tests were observed.

### Strengths and weaknesses of the review

An important strength of this study was its comprehensive search strategy using several search engines to identify any unpublished studies in the form of conference abstracts or proceedings. Screening, study selection, quality assessment and data extraction were undertaken independently and reproducibly by two reviewers, as such human error should be limited. The problem of missing data was reduced by contacting the authors of the publications. In accordance with the study guidelines, potential publication bias and heterogeneity was explored [15, 84]. Evaluation of level of publication bias was not formally carried out in the study; however, the risk of this bias was reduced by not restricting the search to any language. In addition, we contacted experts for information on additional studies. Another strength of this review is that RT-PCR assay has comparably high sensitivity with paucibacillary specimens and high throughput capacities.

This review does, however, have some limitations in assessing issues such as cost-effectiveness and the net effect of RT-PCR assay on clinical care and patient outcomes. Also, because of poor reporting, an analysis of the effect of factors such as laboratory infrastructure was not possible. Secondly, empirical evidence suggests that studies with significant or favourable results are more likely to be published than those with non-significant or unfavourable results [85]. In addition, literature search strategies are inherently imperfect and studies can be missed, it is therefore possible that a proportion of such studies with non-significant or unfavourable results may have been missed. Other limitations are conflicts of interest of study authors particularly from industry supported studies and fully keeping up to date with the primary studies in this rapidly evolving field.

Given that RT-PCR assays in this review cover a wide range of different target genes and procedures, it is not possible to recommend any one over another owing to a lack of direct test comparisons. Our findings should be interpreted in the context of the quality of studies and reporting and variability in study quality. Diagnostic studies in general [86] and TB diagnostic studies in particular [87] seem to be beset by these problems.

### Implications for research and clinical practice

Current evidence suggests a potential role for RT-PCR assay in confirming a diagnosis of TB. It offers an alternative robust approach to detect MTB in paucibacillary EPTB samples that provides rapid results with good diagnostic accuracy. The results of this assay type should be interpreted in parallel with clinical findings and the results of conventional tests; but the assay contributes significantly for an early diagnosis and exerts an impact on clinical management and control of TB. Our findings do not support the use of this assay type for excluding diagnosis of tuberculosis as standalone test.

For EPTB, clinical judgement has both poor sensitivity and specificity. The NICE guidelines recommend the use of culture, histology and/or chest X-ray for patients with non-respiratory TB [88]. Consequently, outcomes of a negative smear for acid-fast bacilli, lack of granulomas on histopathology and failure to culture MTB do not exclude the diagnosis of EPTB; RT-PCR assay has proved to be a novel diagnostic modality in varied forms of EPTB. This review suggests RT-PCR assay can be of help as a most specific test in a ‘rule-in decision’ for MTB detection.

The reliability of RT-PCR to confirm an early diagnosis of TB meningitis and abdominal TB has been well established when smear and culture tests are rarely positive [89, 90]. It has also proved useful for an early diagnosis of osteoarticular TB in tissue samples and that can help to start timely, appropriate anti-tubercular therapy (ATT) [91] and prevent progression to irreversible tissue changes. Due to small sample volumes available, irregular dispersion of MTB in specimens both viable and non-viable, RT-PCR has aided in detecting MTB compared with conventional tests from an array of different cases of EPTB such as pericardial tuberculosis, disseminated/miliary tuberculosis, thyroid tuberculosis, ocular tuberculosis, tuberculous mastitis and others [90].

Future studies should compare commercialised RT-PCR assays to determine their diagnostic accuracy. The use of guidelines such as the Standards for Reporting of Diagnostic Accuracy (STARD) might improve the quality of reporting of primary studies [92]. Further work is required to devise a simple and cost-effective RT-PCR test for an efficient diagnosis of TB that can be used routinely in resource-poor countries.

## Conclusion

According to this review and meta-analysis, RT-PCR assay has a high sensitivity and specificity for PTB with turn-a-round time of 2 h compared with reference culture-based assay that takes between 2 and 10 weeks for detection. Overall, RT-PCR assay accuracy was superior for pulmonary samples (sensitivity 0.82 (95% CI 0.81–0.83); specificity 0.99 (95% CI 0.99–0.99)) as opposed to extra-pulmonary samples (sensitivity 0.70 (95% CI 0.67–0.72); specificity 0.99 (95% CI 0.99–0.99)) possibly due to paucibacillary. The specificity was high for both pulmonary and extra-pulmonary samples indicating that the test should be adopted as the first-line test for ruling in TB infection but may need to be an add-on test to rule out the disease. It offers an alternative robust approach to detect MTB in paucibacillary EPTB samples, showing rapid results with good diagnostic accuracy. The results of this assay should be interpreted in parallel with clinical findings and the results of conventional tests, but the assay may contribute significantly for an early diagnosis and exert an impact on the clinical management and control of TB. The findings do not support the use of this assay for excluding a diagnosis of TB on its own as a standalone test. It offers an incremental benefit as an add-on test to other investigations. RT-PCR assays, combining amplification and detection in a single run, seem to offer advantages over conventional assays including the reference standard.

From the data of investigations of heterogeneity, factors such as RT-PCR assay types (CobasTaqMan, Light Cycler, Cepheid and others) and RT-PCR assay target sequence genes (IS6110, 16SRNA and other genes) were considered to have influenced the accuracy estimates.

It is anticipated that our findings will aid healthcare practitioners and policymakers in adopting the use of this assay on a routine basis. Most importantly, this can be as a point-of-care-test which will help in the global control of MTB, particularly in developing countries with a high burden of the disease.

## Additional files

Additional file 1: PRISMA checklist. (DOCX 17 kb)

Additional file 2: Search strategy. (DOC 28 kb)

Additional file 3: Quality assessment of diagnostic accuracy. (DOC 49 kb)

Additional file 4: Definition of statistical parameters. (DOC 39 kb)

Additional file 5: Figures of Subgroup analyses. (DOC 1822 kb)

## Abbreviations

ATT: Anti-tubercular therapy
CIDG SR: Cochrane Infectious Diseases Group Specialised Register
CPCI-S: Conference Proceedings Citation Index- Science
DNA: Deoxyribonucleic acid
EPTB: Extra-pulmonary tuberculosis
HIV: Human immunodeficiency virus
IGRAs: Interferon-Gamma Release Assays
IUALTD: International Union Against Tuberculosis and Lung Disease
MTB: *Mycobacterium tuberculosis*
NAATs: Nucleic Acid Amplification Tests
NICE: National Institute for Health and Care Excellence
PRISMA: Preferred Reporting Items for Systematic Reviews and Meta-Analyses
PTB: Pulmonary tuberculosis
QUADAS-2: Quality Assessment of Diagnostic Accuracy Studies-2
RT-PCR: Real-time polymerase chain reaction
STARD: Standards for Reporting of Diagnostic Accuracy
TB: Tuberculosis
TRIP: Turning research into practice
TST: Tuberculin skin test
WHO: World Health Organization
