# Supplementary material for: Effectiveness of real-time polymerase chain reaction assay for the detection of Mycobacterium tuberculosis in pathological samples: a systematic review and meta-analysis
Source: Syst Rev. 2017 Oct 25;6:215. doi: 10.1186/s13643-017-0608-2 (PMC5657121; doi:10.1186/s13643-017-0608-2)
Supplement: Supplementary file 2 — Search strategy. (DOC 28 kb) [file 13643_2017_608_MOESM2_ESM.doc]

**Additional file 2: Search strategy**

**MEDLINE via PUBMED Search Strategy:**

**Nov 2016:**

Search terms included ("tuberculosis"[MeSH Terms] OR "tuberculosis"[All Fields]) OR (extrapulmonary[All Fields] AND ("tuberculosis"[MeSH Terms] OR "tuberculosis"[All Fields]))) AND "Real-time polymerase chain reaction"[All Fields]) OR "rt-pcr"[All Fields]) OR "Nucleic Acid Amplification Test"[All Fields]) OR "NAAT"[All Fields]) AND "culture-based media"[All Fields]) OR "liquid media"[All Fields]) AND "solid media"[All Fields]

**Search History**:

**July 2015**

1. Medline; TB.ti,ab; 31,471 results.

2. Medline; tuberculosis.ti,ab; 151,442 results.

3. Medline; exp TUBERCULOSIS/; 162,848 results.

4. Medline; exp MYCOBACTERIUM TUBERCULOSIS/; 38,636 results.

5. Medline; 1 OR 2 OR 3 OR 4; 221,803 results.

6. Medline; (real-time AND PCR).ti,ab; 76,991 results.

7. Medline; (Real-time AND polymer*).ti,ab; 31,904 results.

8. Medline; exp REAL-TIME POLYMERASE CHAIN REACTION/; 27,814 results.

9. Medline; (Culture-based AND assay*).ti,ab; 664 results.

10. Medline; 6 OR 7 OR 8 OR 9; 111,331 results.

11. Medline; 5 AND 10; 826 results

**Search History**:

**July 2015**

1. EMBASE; TB.ti,ab; 39,743 results.

2. EMBASE; tuberculosis.ti,ab; 161,051 results.

3. EMBASE; exp TUBERCULOSIS/; 193,977 results.

4. EMBASE; exp MYCOBACTERIUM TUBERCULOSIS/; 52,324 results.

5. EMBASE; 12 OR 13 OR 14 OR 15; 247,183 results.

6. EMBASE; (real-time AND PCR).ti,ab; 114,589 results.

7. EMBASE; (Real-time AND polymer*).ti,ab; 37,196 results.

8. EMBASE; exp REAL-TIME POLYMERASE CHAIN REACTION/; 118,341 results.

9. EMBASE; (Culture-based AND assay*).ti,ab; 884 results.

10. EMBASE; 17 OR 18 OR 19 OR 20; 169,510 results.

11. EMBASE; 16 AND 21; 1,313 results.
