# Supplementary material for: Effectiveness of real-time polymerase chain reaction assay for the detection of Mycobacterium tuberculosis in pathological samples: a systematic review and meta-analysis
Source: Syst Rev. 2017 Oct 25;6:215. doi: 10.1186/s13643-017-0608-2 (PMC5657121; doi:10.1186/s13643-017-0608-2)
Supplement: Supplementary file 3 — Quality assessment of diagnostic accuracy. (DOC 49 kb) [file 13643_2017_608_MOESM3_ESM.doc]

**Additional file 3**: Quality assessment of diagnostic accuracy

studies-2 tool

QUADAS-2 tool: Risk of bias and applicability judgments

| Domain 1: Patient selection | |
| --- | --- |
| 1. Risk of bias |  |
| Describe methods of patient selection: | |
| - Was a consecutive or random sample of patients enrolled? | Yes/No/Unclear |
| - Was a case-control design avoided? | Yes/No/Unclear |
| - Did the study avoid inappropriate exclusions? | Yes/No/Unclear |
| Could the selection of patients have introduced bias? | RISK: LOW/HIGH/UNCLEAR |
| 1. Concerns regarding applicability |  |
| Describe included patients (prior testing, presentation, intended use of index test and setting): | |
| Is there concern that the included patients do not match the review question? | CONCERN: LOW/HIGH/UNCLEAR |
| Domain 2: Index test(s) *(if more than 1 index test was used, please complete for each test)* | |
| 1. Risk of bias |  |
| Describe the index test and how it was conducted and interpreted: | |
| - Were the index test results interpreted without knowledge of the results of the reference standard? | Yes/No/Unclear |
| - If a threshold was used, was it pre-specified? | Yes/No/Unclear |
| Could the conduct or interpretation of the index test have introduced bias? | RISK: LOW/HIGH/UNCLEAR |
| 1. Concerns regarding applicability |  |
| Is there concern that the index test, its conduct, or interpretation differ from the review question? | CONCERN: LOW/HIGH/UNCLEAR |
| Domain 3: Reference standard | |
| 1. Risk of bias |  |
| Describe the reference standard and how it was conducted and interpreted: | |
| - Is the reference standard likely to correctly classify the target condition? | Yes/No/Unclear |
| - Were the reference standard results interpreted without knowledge of the results of the index test? | Yes/No/Unclear |
| Could the reference standard, its conduct, or its interpretation have introduced bias? | RISK: LOW/HIGH/UNCLEAR |
| 1. Concerns regarding applicability |  |
| Is there concern that the target condition as defined by the reference standard does not match the review question? | CONCERN: LOW/HIGH/UNCLEAR |
| Domain 4: Flow and timing | |
| 1. Risk of bias |  |
| Describe any patients who did not receive the index test(s) and/or reference standard or who were excluded from the 2x2 table (refer to flow diagram):  Describe the time interval and any interventions between index test(s) and reference standard: | |
| - Was there an appropriate interval between index test(s) and reference standard? | Yes/No/Unclear |
| - Did all patients receive a reference standard? | Yes/No/Unclear |
| - Did patients receive the same reference standard? | Yes/No/Unclear |
| - Were all patients included in the analysis? | Yes/No/Unclear |
| Could the patient flow have introduced bias? | RISK: LOW/HIGH/UNCLEAR |
