# Supplementary material for: Effectiveness of real-time polymerase chain reaction assay for the detection of Mycobacterium tuberculosis in pathological samples: a systematic review and meta-analysis
Source: Syst Rev. 2017 Oct 25;6:215. doi: 10.1186/s13643-017-0608-2 (PMC5657121; doi:10.1186/s13643-017-0608-2)

**Additional file 4:** Definition of statistical parameters

**AUC**: The area under the (regression) curve also measures the overall accuracy of diagnostic tests. If the AUC is 100%, then the test differentiates perfectly between diseased and non-diseased individuals. An AUC of 50% indicates a poor diagnostic accuracy.

**DOR**: This was calculated by positive likelihood ratio/negative likelihood ratio or *[sensitivity/ (1-specificity)]/ [(1-sensit​ivity)/ specificity].* DOR is a measure of the overall diagnostic power of a test. A high DOR implies that the test shows good diagnostic accuracy in all patients; whereas a DOR of 1 would indicate that the test cannot discriminate between people with and without disease.

**I^2^**: A statistic describing the proportion of total variation in study estimates that is due to heterogeneity. Values greater than 50% suggest greater heterogeneity between the studies. If the I^2^ statistic suggests significant heterogeneity between the studies then the reasons for such differences can be examined by relating study level co-variates i.e. type of reference test, prevalence, type of PCR or other methodological features.

**Negative likelihood ratio*:*** ratio of the proportion that test negative amongst those that have the target condition compared to the proportion that test negative amongst those who do not have the target condition.

**Positive likelihood ratio*:*** ratio of the proportion that test positive amongst those that have the target condition compared to the proportion that test positive amongst those who do not have the target condition.

**Q**: Is the intercept of the SROC and the anti-diagonal line through the unit square i.e. the point of the curve in which sensitivity equals specificity. Q estimates the overall accuracy by finding where sensitivity and specificity are the same. If the curve is closer to the top left corner, the better the accuracy. The higher the Q, the more accurate is the test. However, a high Q is desirable in tests where high sensitivity and high specificity are equally important. If, however, when either the sensitivity or the specificity is more important than one of them, then Q does not address the clinical usefulness of the test.

**Receiver characteristic operating curve (ROC)*:*** the sensitivity and specificity of a test vary depending on the threshold value chosen. The ROC curve describes the trade-off between sensitivity and specificity as the threshold changes.

**Sensitivity*:*** proportion that test positive amongst those having the target condition.

**Specificity*:*** proportion that test negative amongst those without the target condition.

**Summary receiver characteristic operating curve (SROC)**: This displays each study's sensitivity and specificity estimates within the ROC space. A regression curve is fitted through the distribution of pairs of sensitivity and specificity. A shoulder-like curve indicates that the variability between studies may be due to the threshold effect (i.e. variation in cut-off values used across studies) and that an underlying common DOR exists that does not change with the threshold. A non-shoulder-like curve shows that sensitivity and specificity are not correlated.

**Threshold*:*** A value above or below which a test result is considered positive.

**Moses-Littenberg statistical modelling of ROC curves**


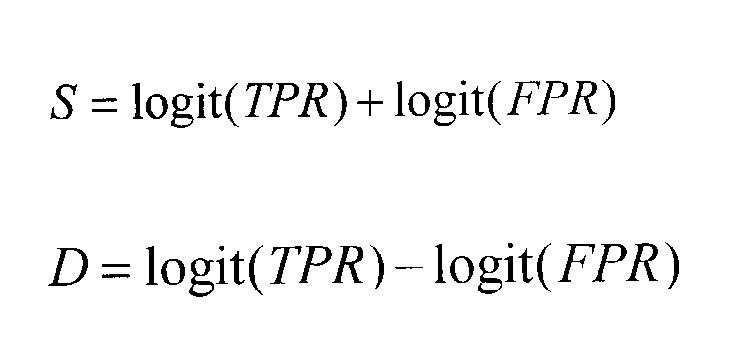

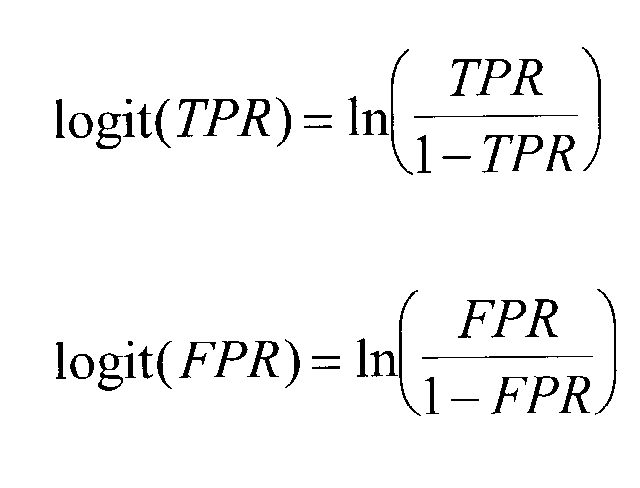

Supplement: Supplementary file 4 — Definition of statistical parameters. (DOC 39 kb) [file 13643_2017_608_MOESM4_ESM.doc]
