# Supplementary material for: Effectiveness of real-time polymerase chain reaction assay for the detection of Mycobacterium tuberculosis in pathological samples: a systematic review and meta-analysis
Source: Syst Rev. 2017 Oct 25;6:215. doi: 10.1186/s13643-017-0608-2 (PMC5657121; doi:10.1186/s13643-017-0608-2)
Supplement: Supplementary file 5 — Figures of Subgroup analyses. (DOC 1822 kb) [file 13643_2017_608_MOESM5_ESM.doc]

**Additional file 5:** Figures of Subgroup analyses


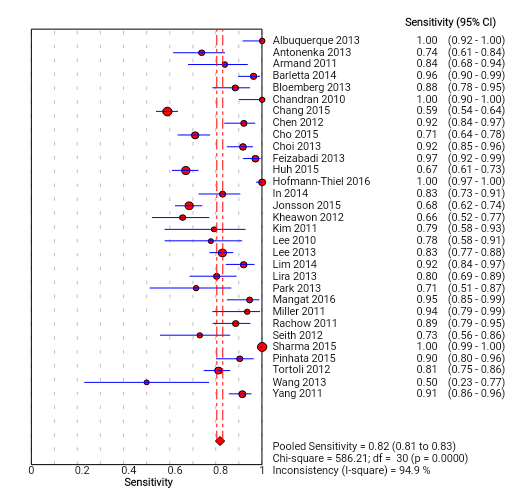


**Figure S6** Forest plot estimates of the pooled sensitivity for PTB


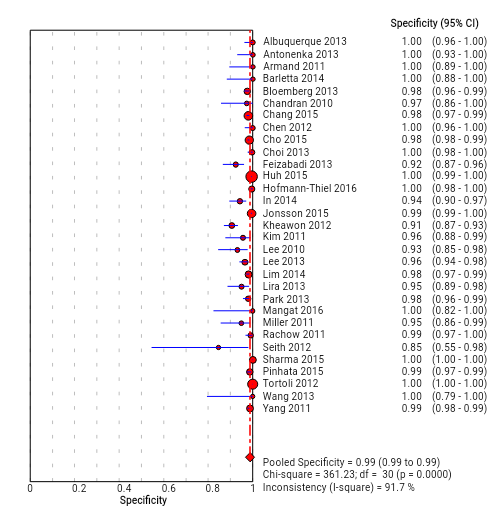


**Figure S7** Forest plot estimates of the pooled specificity for PTB


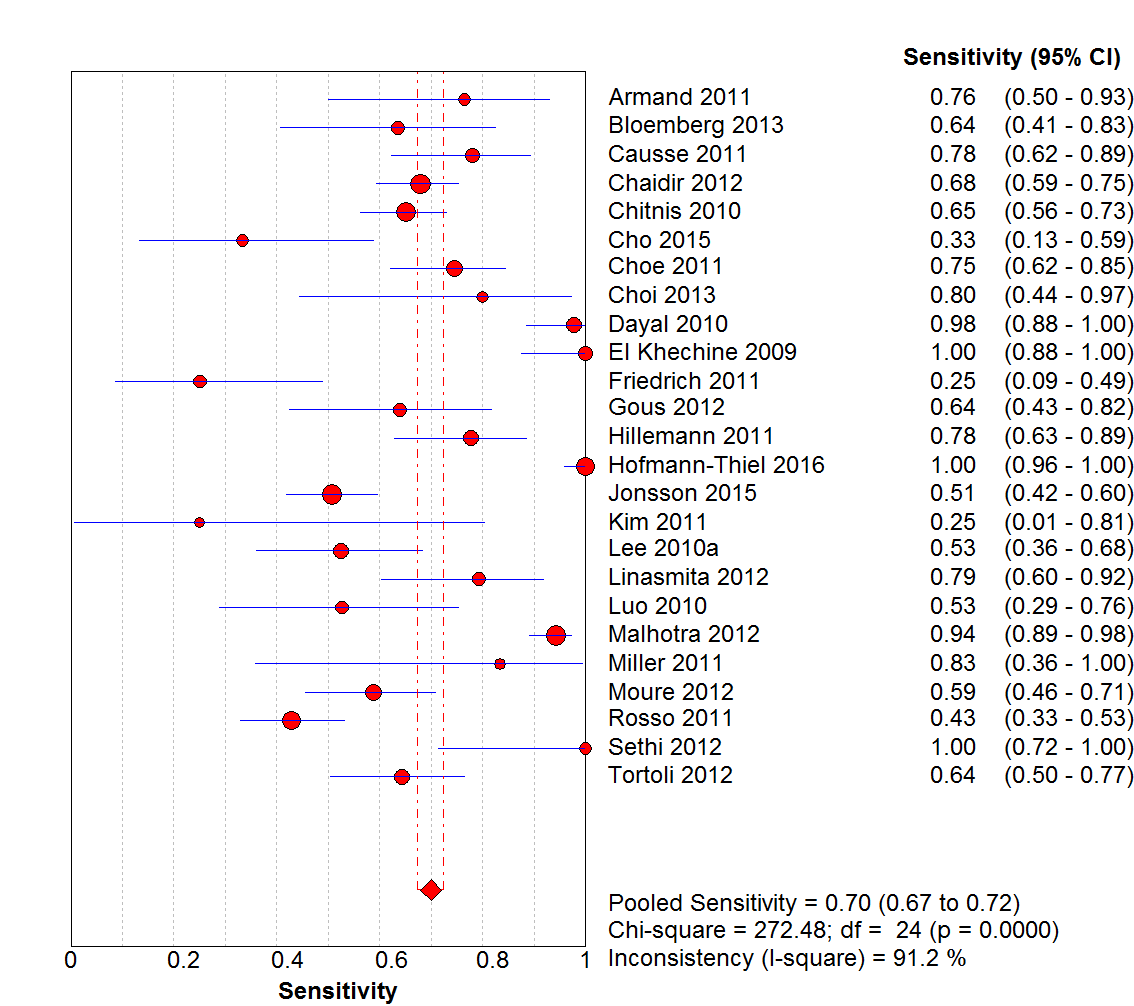


**Figure S8** Forest plot estimates of the pooled sensitivity for EPTB


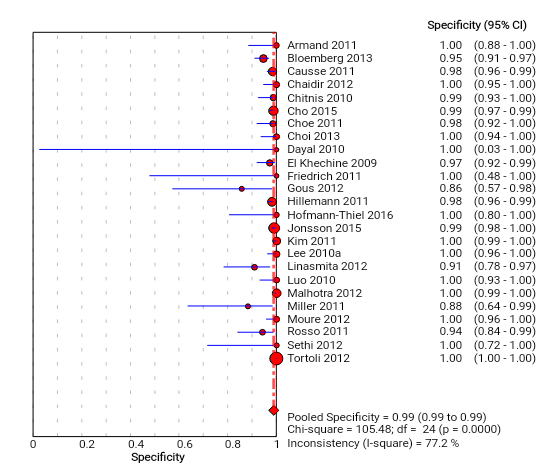


**Figure S9** Forest plot estimates of the pooled specificity for EPTB


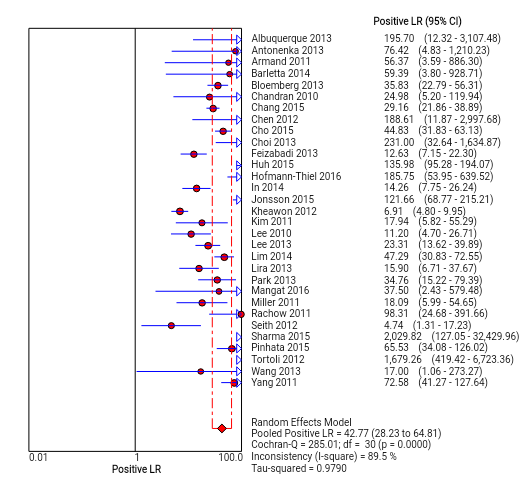


**Figure S10 (a, b, c &d)** Forest plot estimates of pooled PLR, NLR, DOR, and SROC for PTB


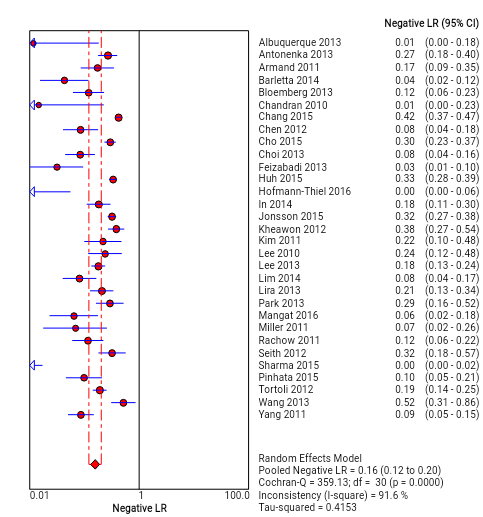


**Figure S10 b**


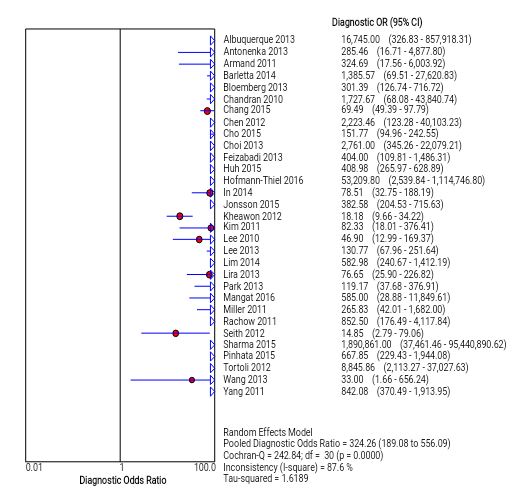


**Figure S10 c**


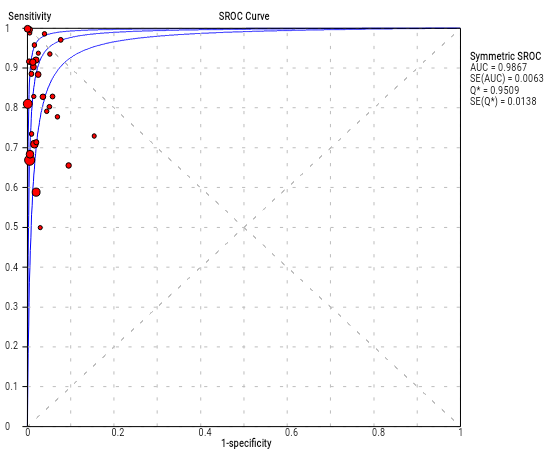


**Figure S10 d**


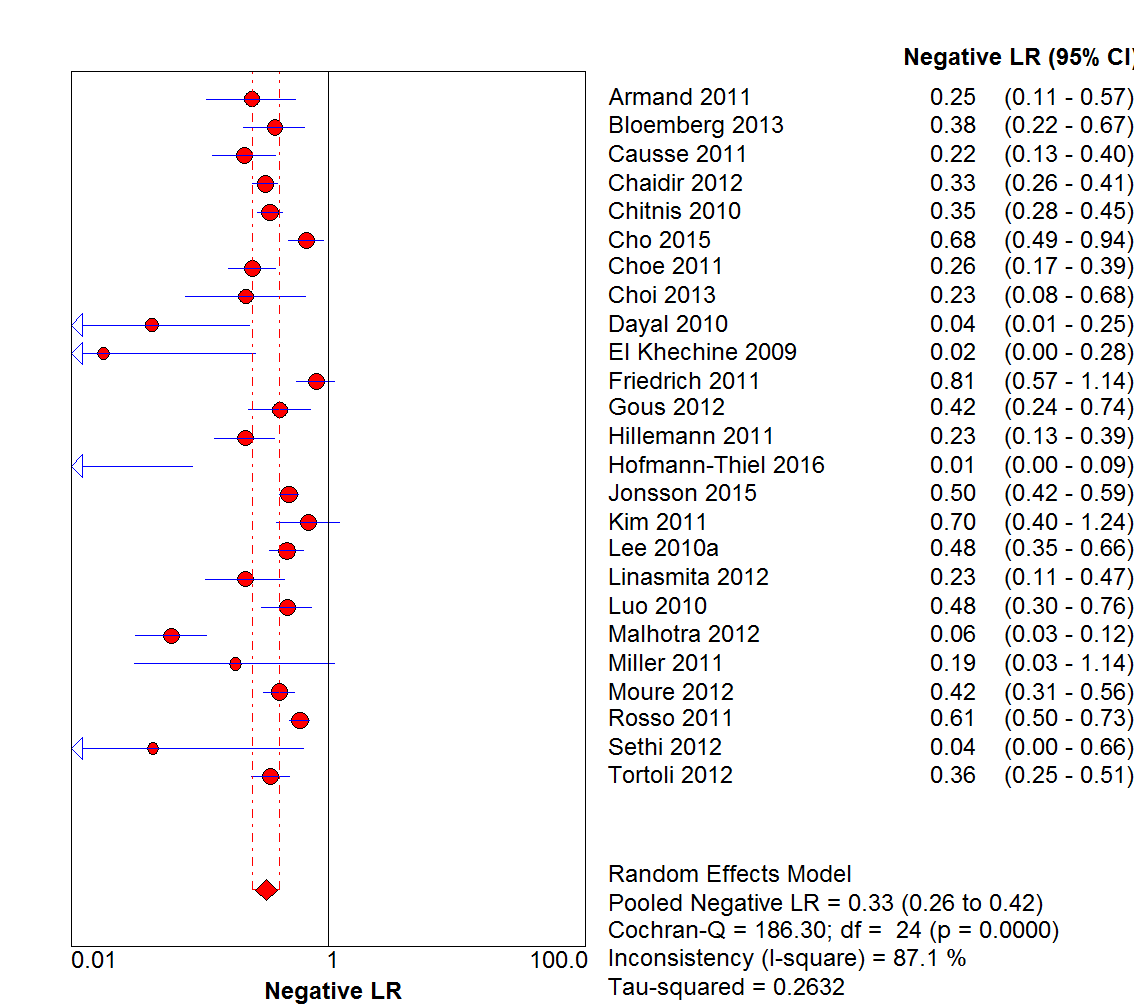


**Figure S11 (a, b, c &d)** Forest plot estimates of pooled PLR, NLR, DOR, and SROC for EPTB


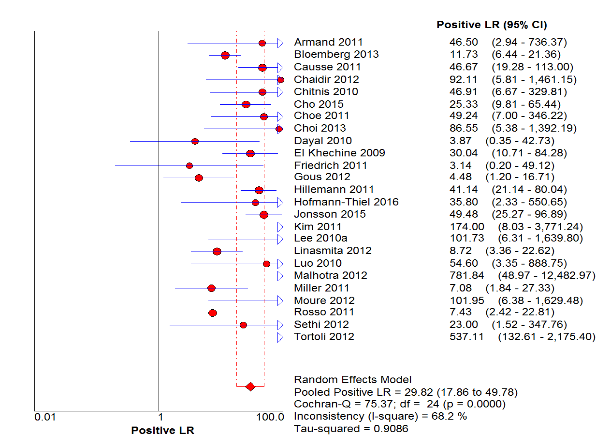


**Figure S11 b**


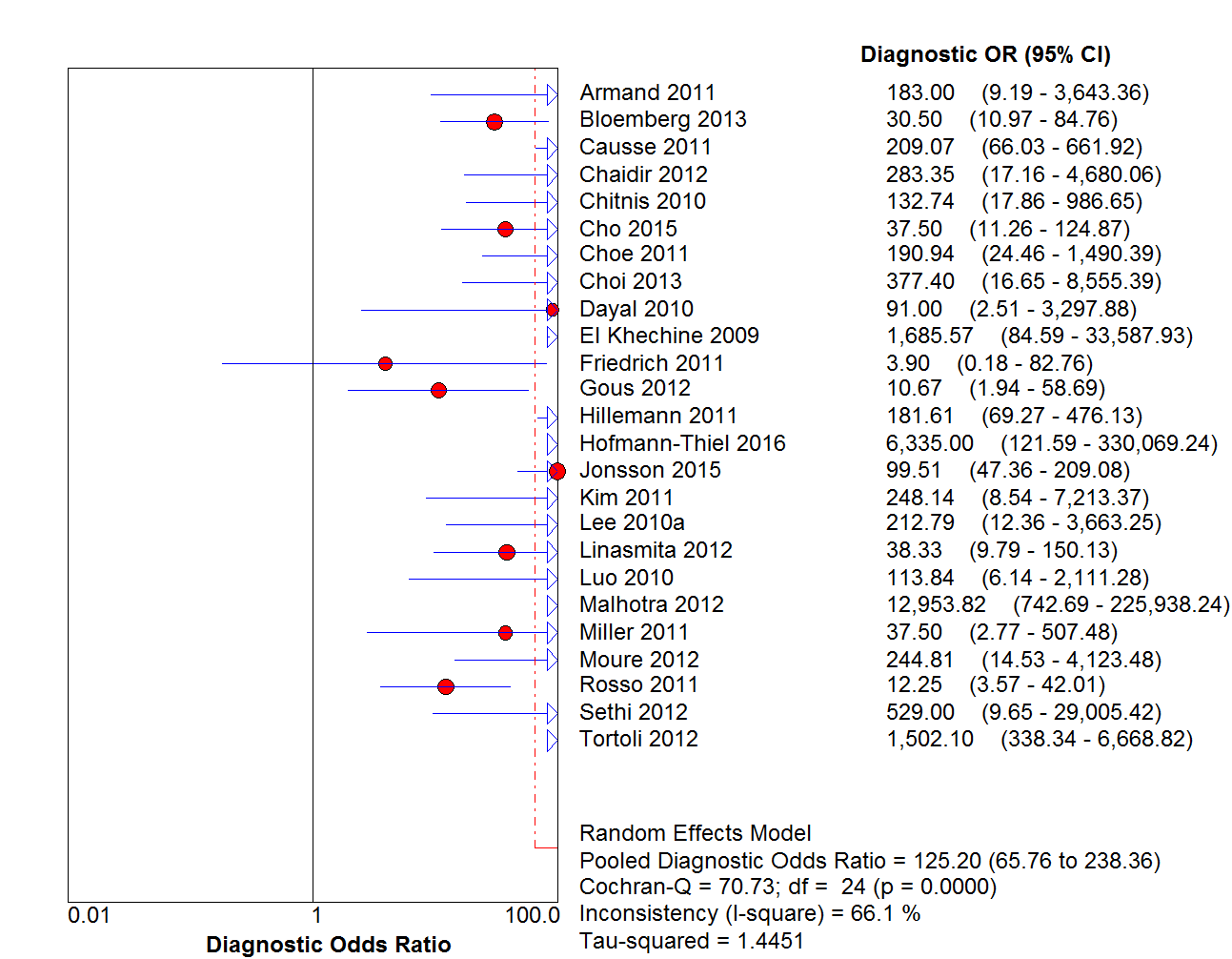


**Figure S11 c**


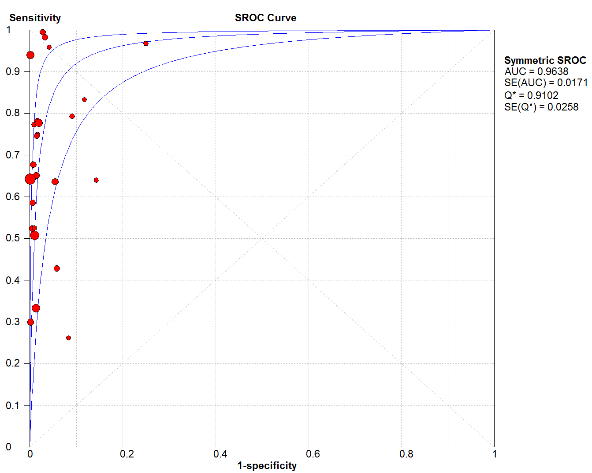


**Figure S11 d**


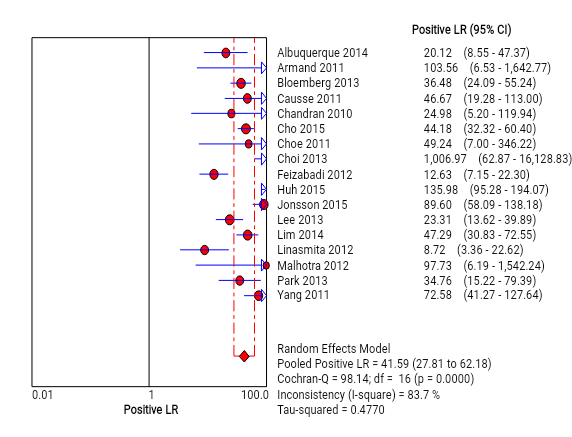


**Figure S12 (a, b, c &d)** RT-PCR assay type: Cobas TaqMan


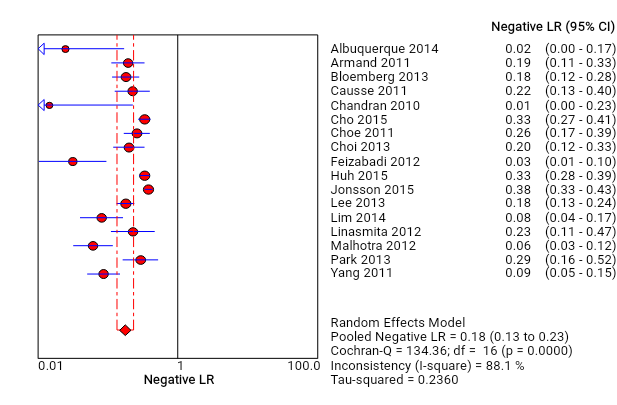


**Figure S12 b**


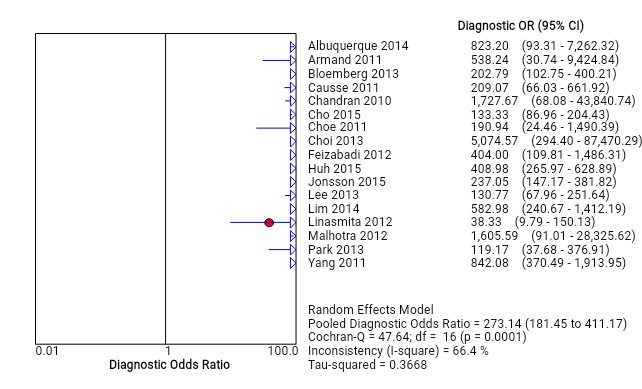


**Figure S12 c**


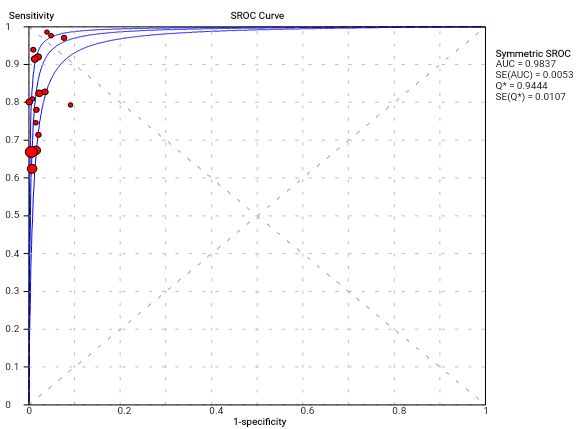


**Figure S12 d**


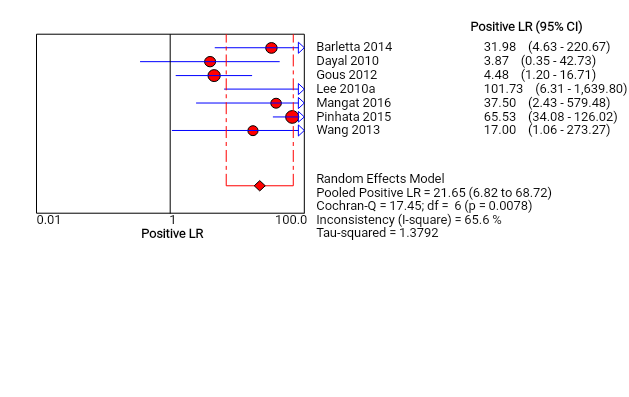


**Figure 13 (a, b, c &d)** **RT**-PCR assay type: Light Cycler


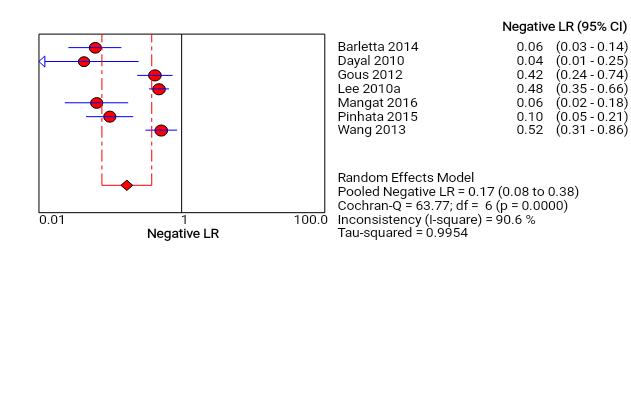


**Figure 13 b**


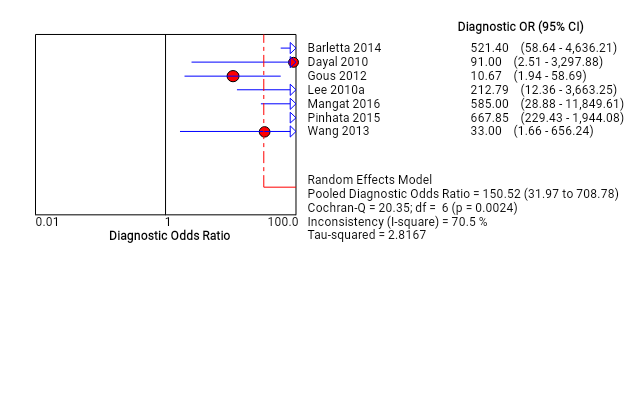


**Figure 13 c**


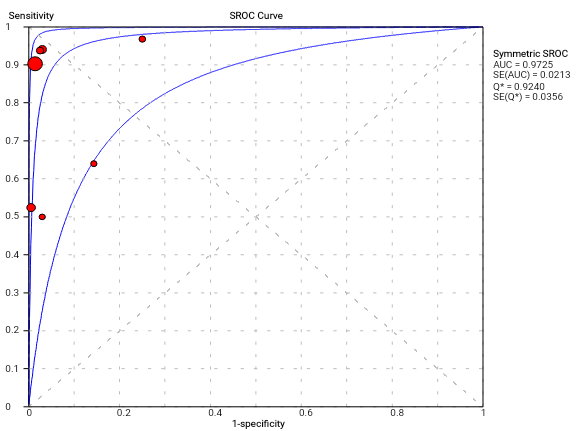


**Figure 13 d**


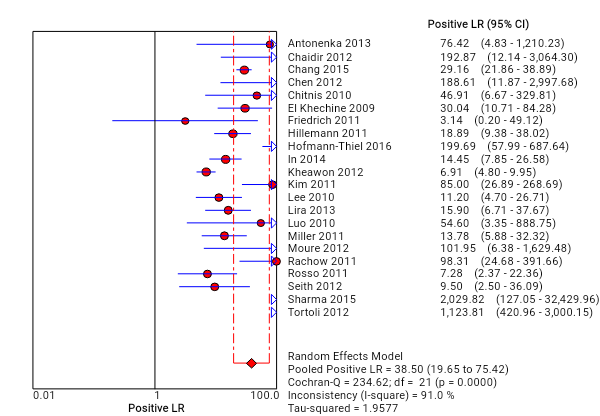


**Figure 14 (a, b, c &d)** RT-PCR assay type: Cepheid and others


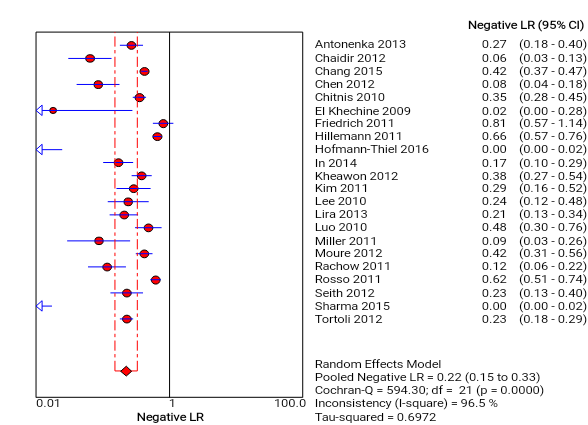


**Figure 14 b**


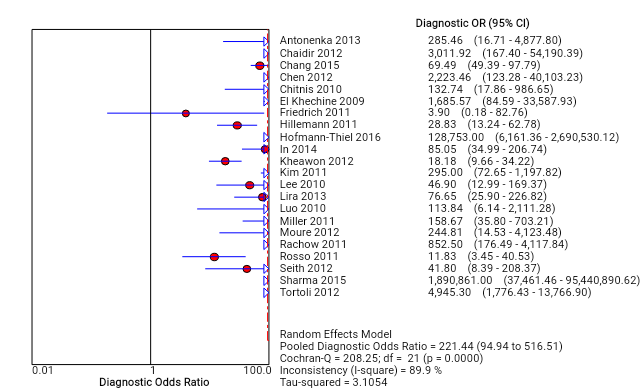


**Figure 14 c**


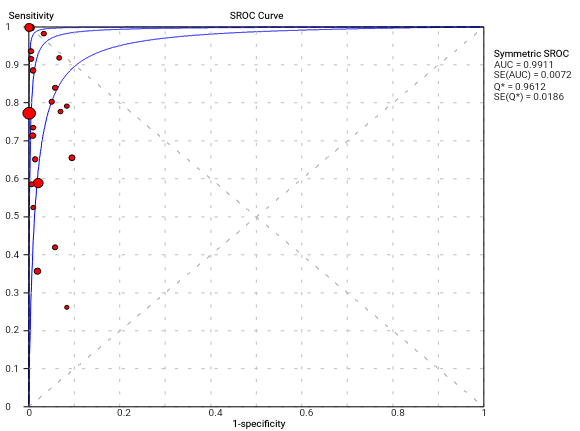


**Figure 14 d**


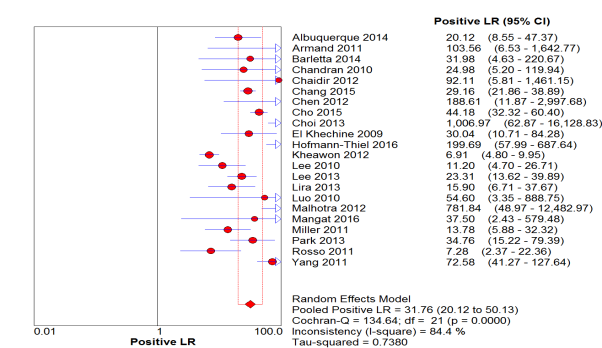


**Figure 15 (a, b, c &d)**  RT-PCR assay target sequence: IS6110 gene


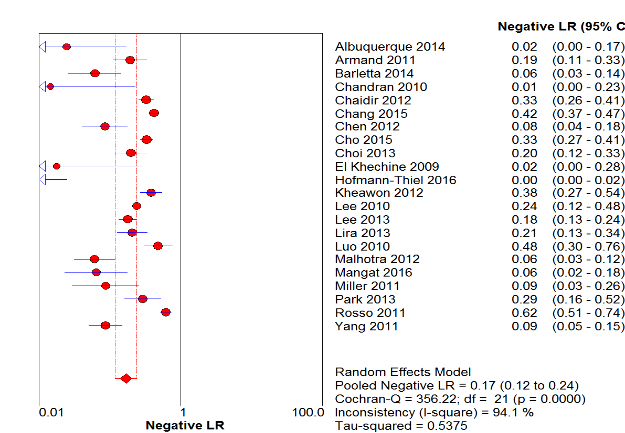


**Figure 15 b**


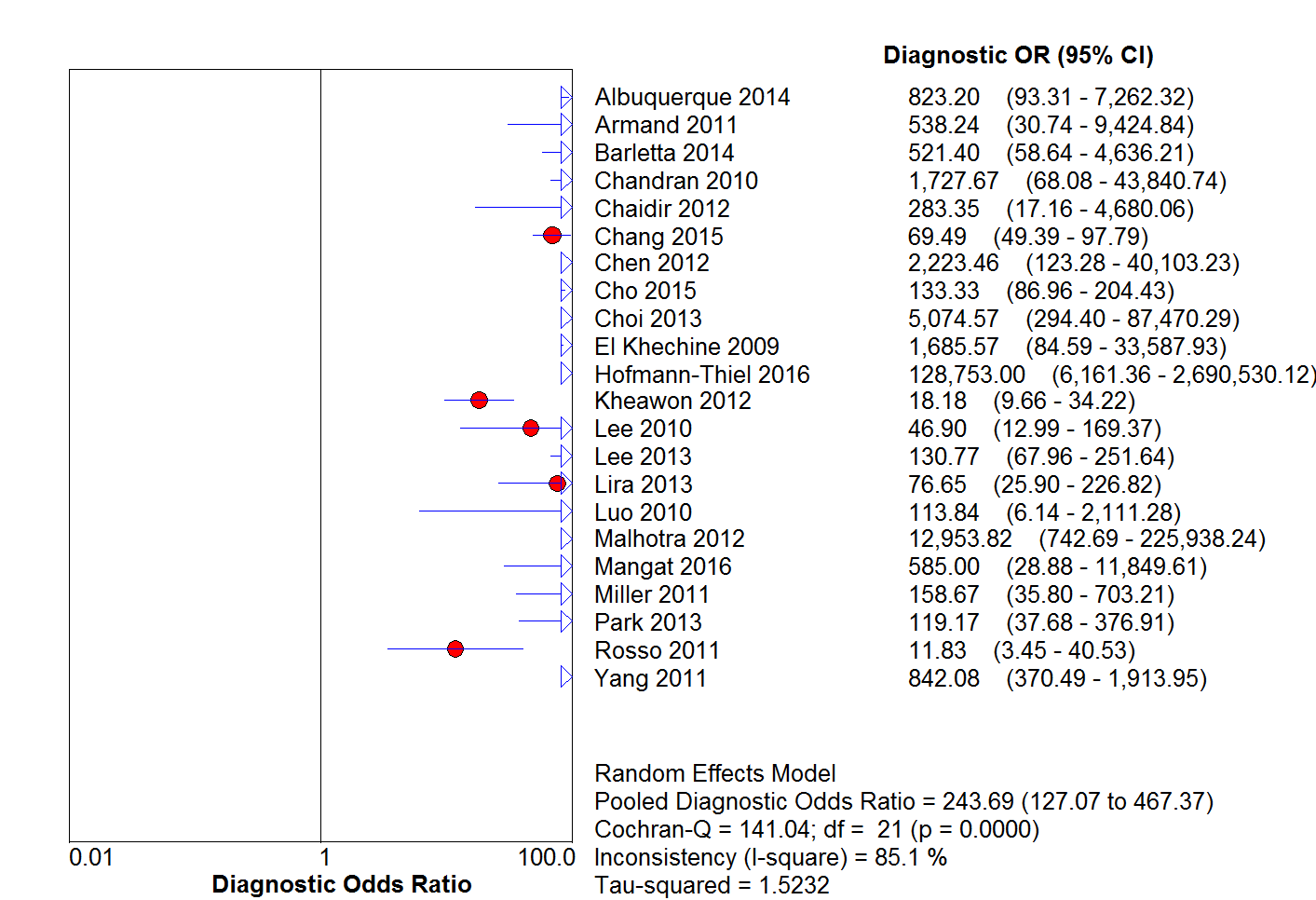


**Figure 15 c**


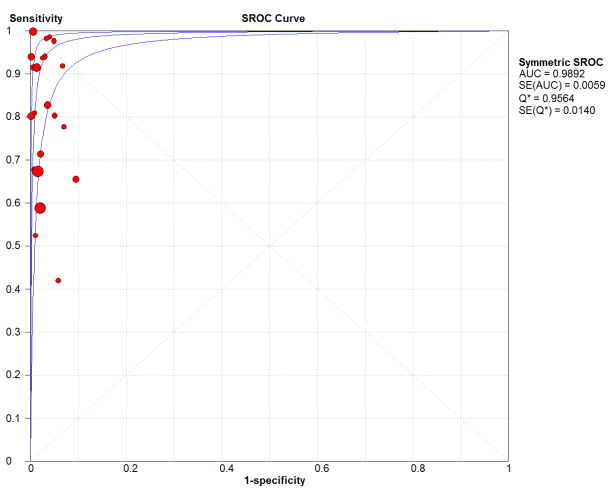


**Figure 15 d**


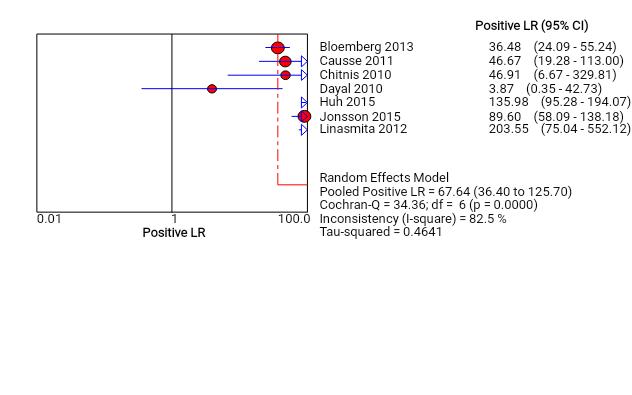


**Figure 16 (a, b, c &d)** RT-PCR assay target sequence: 16S r RNA gene


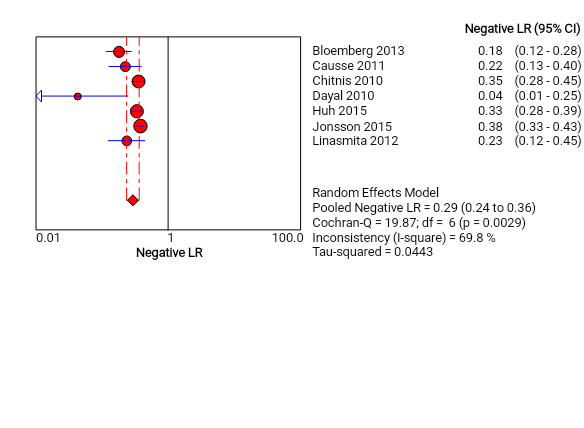


**Figure 16 b**


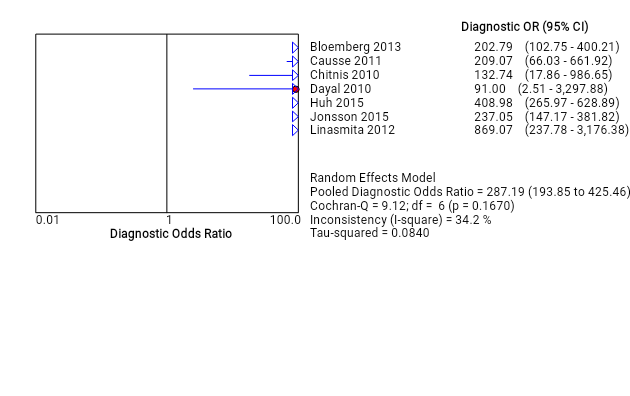


**Figure 16 c**


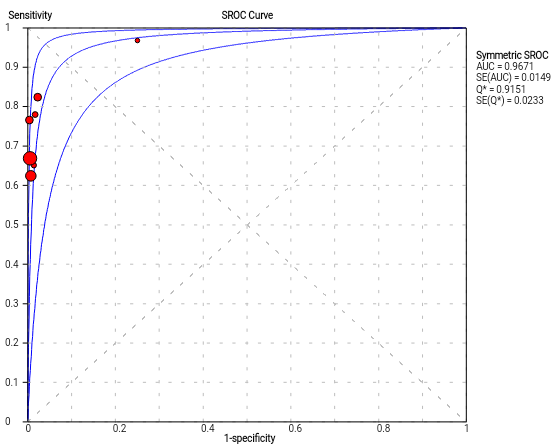


**Figure 16 d**


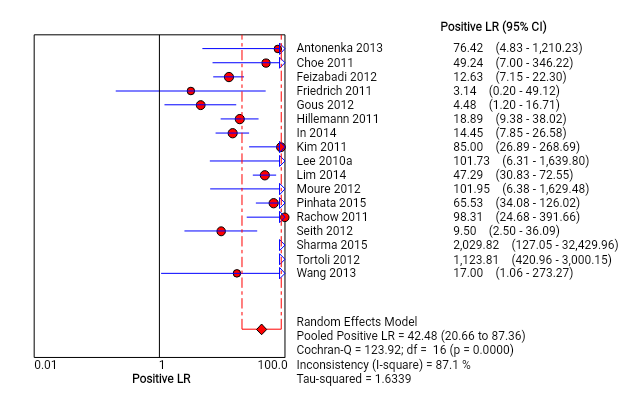


**Figure 17 (a, b, c &d)** RT-PCR target sequences: other genes


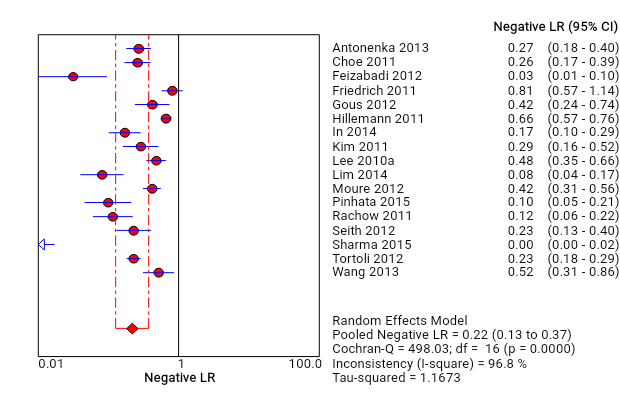


**Figure 17 b**


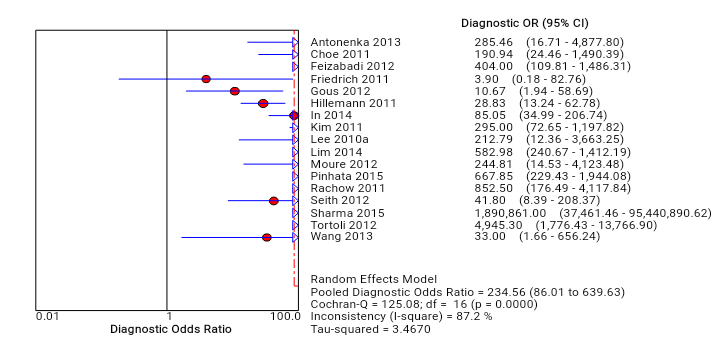


**Figure 17 c**


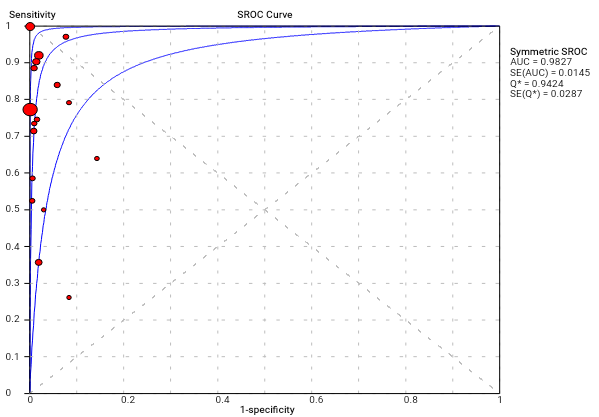


**Figure 17 d**
